# Predicting functions of uncharacterized gene products from microbial communities

**DOI:** 10.1038/s41587-025-02813-7

**Published:** 2025-10-15

**Authors:** Yancong Zhang, Amrisha Bhosle, Sena Bae, Kelly Eckenrode, Xueying Huang, Jingjing Tang, Danylo Lavrentovich, Lana Awad, Ji Hua, Ya Wang, Xochitl C. Morgan, Bin Li, Andy Krueger, Wendy S. Garrett, Eric A. Franzosa, Curtis Huttenhower

**Affiliations:** 1 Shenzhen Branch, Guangdong Laboratory of Lingnan Modern Agriculture, Genome Analysis Laboratory of the Ministry of Agriculture and Rural Affairs, Agricultural Genomics Institute at Shenzhen, Chinese Academy of Agricultural Sciences, Shenzhen, China; 2 Infectious Disease and Microbiome Program, Broad Institute of MIT and Harvard, Cambridge, MA USA; 3 Department of Biostatistics, Harvard T. H. Chan School of Public Health, Boston, MA USA; 4 Harvard Chan Microbiome in Public Health Center, Harvard T. H. Chan School of Public Health, Boston, MA USA; 5 Department of Immunology and Infectious Diseases, Harvard T. H. Chan School of Public Health, Boston, MA USA; 6 Systems, Synthetic, and Quantitative Biology Program, Harvard University, Cambridge, MA USA; 7 Takeda Development Center Americas, Inc., Lexington, MA USA; 8 Department of Medical Oncology, Dana-Farber Cancer Institute, Boston, MA USA

**Keywords:** Protein function predictions, Microbiome, Gene expression, Software, Data integration

## Abstract

The majority of genes in microbial communities remain uncharacterized. Here we develop a method to infer putative function for microbial proteins at scale by assessing community-wide multiomics data. We predict high-confidence functions for >443,000 protein families (~82.3% previously uncharacterized), including >27,000 protein families with weak homology to known proteins and >6,000 protein families without homology. These were drawn from 1,595 gut metagenomes and 800 metatranscriptomes from the Integrative Human Microbiome Project (HMP2/iHMP). Integrating additional information such as sequence similarity, genomic proximity and domain–domain interactions improves performance of the method. Our method’s implementation, FUGAsseM, is generalizable and predicts protein function in both well-studied and undercharacterized communities. FUGAsseM achieves similar levels of accuracy in the context of microbial communities when compared to state-of-the-art approaches designed for application to single organisms while simultaneously providing much greater breadth of coverage. This initial study expands the functional landscape of the human gut microbiome and allows for exploration of microbial proteins in undercharacterized communities.

## Abstract (web summary)

FUGAsseM predicts protein function in microbiomes using coexpression patterns from metatranscriptomes and diverse community-wide data.

## Main

Microbial proteins represent the entire span of enzymatic, structural and other molecular functions (MFs) necessary across the tree of life, such as metabolizing host-inaccessible dietary components^1^, producing immunomodulatory small molecules^2^ and driving biogeochemical cycling^3–5^. However, even within the human gut microbiome—arguably the best-characterized human microbial community habitat—up to 70% of proteins are uncharacterized^6,7^. The environmental and health relevance of this functional ‘dark matter’ combined with its tremendous scale (for example, several million genes across the diversity of human gut microbiomes^8,9^) necessitates a scalable approach for predicting protein functions in microbial communities^10–12^. Although culture-based and other in vitro techniques provide the gold standard for functional insights^13,14^, it is challenging to rapidly characterize most microorganisms using cultivation in settings similar to their natural environments, let alone in a sufficiently high-throughput manner^15^. Similarly, experimental characterization of proteins is undeniably a labor-intensive, time-consuming and expensive process that cannot approach the scale of thousands of new protein families per year^16^.

Computational strategies for protein function prediction have a long history, especially in microbial isolate genomes^17–19^, and they have gained popularity with the exponential growth of sequence data^20–22^. Such methods typically integrate multiple data types from individual organisms, including sequence similarity^23–26^, genomic context^27,28^, evolutionary relationships^29,30^, protein structure^31^, protein–protein interactions^32–34^, gene expression^35,36^ or an ensemble of these data types^37–39^. Direct application of single-organism methods to communities is greatly limited by the prevalence of novel sequences lacking sequence similarity to known proteins, as well as the gap between shotgun metagenomes (which provide only DNA) and other functional assays. This limits the knowledge of genomic context, protein structures, protein–protein interactions or expression in most communities, making systematic prediction of protein functions for microbial communities challenging.

Given the success of high-throughput sequencing technologies for microbial community characterization, metatranscriptomics (MTX) is becoming an increasingly practical way to profile whole-community RNA to provide microbial functional information in situ^40^. Considering that transcriptional profiles of microorganisms frequently differ between isolate culture and natural community settings^41^, MTX provides a crucial way to link genetic potential (gene carriage and abundance) to functional activity in human^42–44^ and other environmental microbiomes^5,45,46^. Genes with similar functions tend to be coexpressed^47,48^, indicating that they are active in the same biological processes (BPs). Therefore, coexpression patterns have been leveraged for protein function prediction in single-organism settings^36,49,50^. Recently, this approach was applied to infer functions of unknown genes in marine microbial communities^5^. Aside from this exploratory study, however, which did not further develop or investigate the methodology itself, automated function prediction for microbial communities has been surprisingly unexplored.

To address the dramatic undercharacterization of microbial community gene products, we developed a method to systematically predict protein function in microbiomes by leveraging coexpression patterns from metatranscriptomes, along with other types of community-wide evidence such as genomic proximity, sequence similarity and domain–domain interactions of proteins assembled from microbial communities. We use a two-layered random forest (RF) classifier system to assign target proteins to functions through ‘guilt-by-association’ learning functional association networks among microbial proteins. For a given function, an individual RF classifier is trained for each type of association evidence to assign unannotated proteins to that function on the basis of their associations with annotated proteins, forming the first layer of machine learners. In the second layer, an ensemble RF classifier integrates the per-evidence prediction confidence scores from the first layer to produce a single combined confidence score, adjusting evidence weighting per function to capture biological trends and enhance prediction accuracy. The resulting method yields comparably or more accurate predictions than state-of-art single-organism methods while also achieving far greater sensitivity among diverse organisms found only in community data. When applying our method to the Integrative Human Microbiome Project (HMP2/iHMP)^44^, we annotated >443,000 previously uncharacterized protein families with Gene Ontology (GO)^51^ terms (including >33,000 novel protein families that lack notable sequence homology to known proteins) with high-confidence function predictions. In this process, both well-studied and less studied gut taxa were further characterized with increased functional diversity. An open-source implementation of this method (FUGAsseM, function predictor of uncharacterized gene products by assessing high-dimensional community data in microbiomes)^52^, along with documentation, is available online (http://huttenhower.sph.harvard.edu/fugassem). Our work predicts functions of uncharacterized gene products in microbial communities and provides methodology supporting future microbial community-based function prediction.

## Results

### MTX-based coexpression patterns capture comprehensive functional activity in microbial communities

**Fig. 1:**
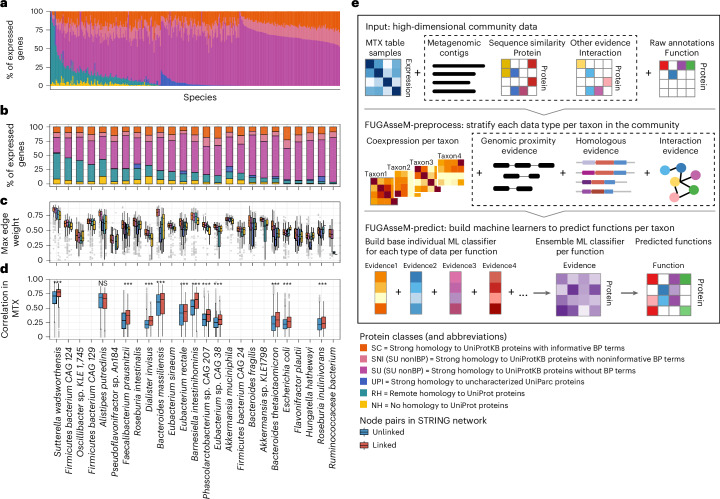
Metatranscriptomes encode functional relationships and can be used for predicting functions of uncharacterized proteins in microbial communities. **a**, Protein families from HMP2 gut metatranscriptomes are dominated by uncharacterized proteins. **b**, Among the top 25 species with the greatest number of novel proteins, the uncharacterized proteins are ubiquitous even in the pangenomes of otherwise characterized species. **c**, Uncharacterized proteins are often highly correlated with characterized proteins in species-stratified MTX and the correlation patterns vary over species (*n* = 44,327 total genes among species). ‘Maximum (Max) edge weight’ is the average of the five strongest correlations to characterized proteins (Supplementary Table 3). Box plots display the median (line at the 50th percentile), interquartile range (IQR; box spanning the 25th to 75th percentiles), whiskers (extending to 1.5 × the IQR) and outliers (values beyond 1.5 × the IQR from the quartiles). **d**, When comparing MTX-based coexpression networks to STRING-based isolate coexpression networks (*n* = 1,035,608 total gene pairs among species), proteins linked in STRING networks tend to have stronger correlations in MTX-based networks but MTX covers many more species. ‘STRING-linked’ protein pairs have linkage scores above the first quartile of the maximum score in STRING; otherwise, they are ‘STRING-unlinked’. Statistical analysis was performed using two-sided unpaired Wilcoxon tests with unadjusted *P* values reported as follows: ***P < 0.001; NS, not significant. The detailed coexpression dataset is provided in Supplementary Table 4. Box plots are displayed as in **c**. **e**, We developed a computational method—FUGAsseM—to predict function assignments for proteins in microbial communities. Using a guilt-by-association approach, FUGAsseM begins by building machine learning (ML) models for each type of community-wide data in a first layer, followed by an ensemble classifier combining data types in a second layer. This results in one prediction confidence per protein per function class. Source data

**Extended Data Fig. 1:**
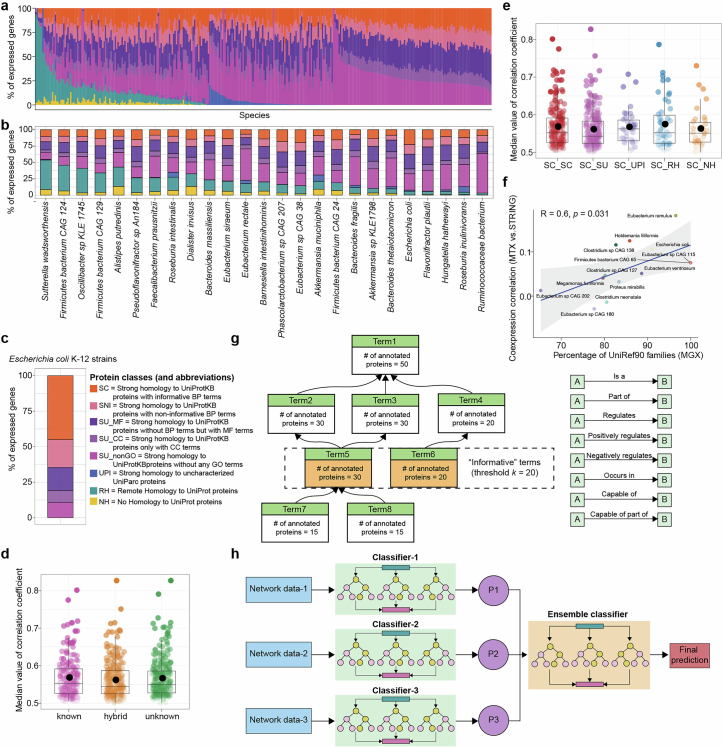
Uncharacterized gut microbial proteins are coexpressed with known proteins and annotated using a two-layer ML model. (**a**) Human gut microbial protein families detected in HMP2 metatranscriptomes were categorized into different levels of under-characterization. (**b**) The top 25 species with the most novel proteins (RH and NH) show diverse protein category composition, with uncharacterized proteins prevalent. (**c**) In contrast to the human gut microbiome, most proteins in *E. coli* K-12 strains are well-characterized. (**d**) Uncharacterized proteins exhibit similar MTX-based coexpression patterns to those of characterized proteins (*n* = 1,543 total gene pairs). ‘known’ indicates transcript correlations among characterized proteins; ‘hybrid’ between characterized and uncharacterized; ‘unknown’ among uncharacterized proteins. Box plots display the median (line at the 50th percentile), interquartile range (box spanning the 25th to 75th percentiles), whiskers (extending to 1.5× IQR), and mean values (dark points). (**e**) Strong transcript correlations were also observed across different characterization levels (*n* = 1,331 total gene pairs); Box plots as in (**d**). (**f**) Network similarity between MTX-based and STRING-based coexpression patterns was significantly correlated with species reference representation. Pearson correlation coefficients (95% CI) and unadjusted *P* values are shown (*n* = 13 species). Reference representation was estimated by the percentage of UniRef90 homologs in HMP2 MGX previously profiled by MetaWIBELE. (**g**) ‘Informative’ GO terms were defined as those with at least a minimum number of annotated proteins, while each child term contained fewer than this threshold. (**h**) FUGAsseM uses a two-layer machine learning architecture. In the first layer (green), random forest classifiers predict functional confidence scores from individual data types (purple). These scores are then integrated by a second-layer ensemble RF classifier (orange), generating a final confidence score (red). Source data

To first assess the relationship between protein function and gene expression in the human microbiome, we screened 800 metatranscriptomes from the HMP2 inflammatory bowel disease (IBD) multiomics database (IBDMDB), which spanned a total of 109 participants with Crohn’s disease (*n* = 52), with ulcerative colitis (*n* = 30) and without IBD (*n* = 27), followed longitudinally for up to 1 year^44^. Specifically, we quantified the expression of the protein families previously profiled from 1,595 metagenomes by MetaWIBELE^7^—a tool that predicts bioactivity from metagenomically assembled protein families (Methods). A total of 582,744 protein families detected in the metatranscriptomes were contributed by 336 species with at least 500 protein families per species (Fig. 1a, Supplementary Table 1 and Extended Data Fig. 1a).

To examine the characterization level of these protein families, we generated a set of ‘informative’ (refs. ^53,54^) BP terms (the largest and most diverse ontology aspect in GO^51^) inspired by single-organism methods^55^. To be included in this set, annotations from child terms were propagated to all ancestor terms within the GO directed acyclic graph (DAG). For a given species, an informative BP term is defined as a term that contains at least a specified number of annotated proteins (taking DAG inheritance into account) while none of its child terms individually reach this threshold (Extended Data Fig. 1g). While the concept is not reliant on a specific threshold number *k*, we used *k* = 20 throughout this work. After applying this criterion to GO protein family annotations within species, the resulting species-specific informative terms were subsequently combined by union to create a single nonredundant set of informative terms across species, ensuring that no term was a parent term of others within the set. In other words, if a term *X* met the *k* = 20 threshold for informativeness in at least one species and was not a parent term of another such term *Y* identified in any species, it was included in the set of informative terms used in this study. All the informative terms used in this study are organized in Supplementary Table 2. According to the novelty categories defined in our previous publication^7^, we classified the aforementioned MTX protein families into ‘SC’ (strong homology to characterized UniProtKB^56^ proteins with informative BP terms), ‘SNI’ (strong homology to characterized UniProtKB proteins with noninformative BP terms), ‘SU’ (strong homology to uncharacterized UniProtKB proteins without any BP terms), ‘UPI’ (strong homology to uncharacterized UniParc proteins), ‘RH’ (remote homology to UniProt proteins) and ‘NH’ (no homology to UniProt proteins) (Methods). SC comprised 83,280 families (14.3% of the total). Conversely, 499,464 families (85.7% of the total) were functionally uncharacterized, including 11.9% SNI, 60.5% SU, 3.6% UPI, 8.0% RH and 1.7% NH.

As a baseline for investigation, we began with pangenomes of common species in the human microbiome. Despite the degree to which these organisms have been well studied as isolates, their pangenomes in typical communities remain predominantly undercharacterized. Overall, 60.5% of the HMP2 protein families were not annotated with any BP terms (that is, SU) in UniProtKB. To further characterize the SU protein families, we included the annotations of MF and cellular component (CC) terms. We stratified SU into ‘SU_MF’ (SU families with MF term annotations in UniProtKB), ‘SU_CC’ (SU families with only CC term annotations in UniProtKB) and ‘SU_nonGO’ (SU families without any GO annotations in UniProtKB). Of the 352,527 SU families, 123,921 and 51,183 families were labeled as SU_MF and SU_CC respectively, while the remaining 77,423 families lacked any GO annotations (Extended Data Fig. 1a). Even in the well-characterized *Escherichia coli* pangenome, only 37.6% protein families were annotated with BP terms and 24.9% were not annotated with any GO terms (Extended Data Fig. 1b). As a positive control, the protein families of *E.* *coli* K-12 strains were well annotated, including 64.6% families with BP terms, 24.6% with either MF or CC terms and only 10.8% without GO annotations (Extended Data Fig. 1c). The predominance of uncharacterized proteins even in the pangenomes of well-studied microorganisms highlights the need for expanded function prediction in microbial communities.

Next, as a first step in assigning putative function predictions, we investigated the degree to which MTX is functionally informative in isolation. Notably, in single organisms, proteins in the same metabolic or regulatory pathway tend to be coexpressed; hence, coexpression indicates functional relatedness and can be used to predict pathway comembership^36,49,50^. In microbial communities, MTX are to date the most widely available whole-community transcriptional data encoding potential functional relatedness. We began with simple MTX coexpression networks (that is, Pearson’s correlation computed over a pair of proteins’ expression values across samples), for which, as expected, both characterized and uncharacterized proteins showed coexpression patterns with strong connection (*R* > 0.5) (Extended Data Fig. 1d). Many uncharacterized protein families were highly correlated with characterized families, where their correlations were comparable to correlations within characterized families (Extended Data Fig. 1e). To further distinguish the legitimate lack of coexpression cases from those where proteins were not well expressed (for example, low prevalence), we defined a set of ‘well-expressed’ proteins (that is, detected in at least 10% of total MTX samples) and measured how the uncharacterized proteins are close to characterized neighbors among these well-expressed proteins in MTX coexpression networks (Methods). Among the top 25 species with the most novel (that is, RH and NH) proteins (Fig. 1b), many well-expressed uncharacterized proteins were strongly correlated with the characterized proteins at the transcriptional level (Fig. 1c and Supplementary Table 3), suggesting their functional relatedness in the gut.

We next sought to compare MTX-based coexpression with isolate-based coexpression using isolate data for HMP2 species from the STRING database^57^. In total, 12 of the top 25 species with the greatest number of novel proteins were available in STRING, in agreement with the observation that a large fraction of species from microbial communities lack corresponding reference isolates (Supplementary Table 4). In most species (11 of 12), STRING-linked proteins showed significantly stronger correlations than STRING-unlinked proteins in MTX-based networks (Mann–Whitney test on difference between average correlation values, *P* < 0.05) (Fig. 1d). Moreover, the coexpression similarity (quantified by Pearson’s correlation) between MTX and STRING networks was itself significantly correlated with the reference representation of HMP2 species (Pearson’s correlation, *R* = 0.6, *P* = 0.031) (Extended Data Fig. 1f). That is, the better characterized a species is, the more similar its MTX-based and STRING-based coexpression networks are. However, many more coexpression relationships were captured by MTX than by isolates; only *E.* *coli* and *Pseudomonas aeruginosa* contained direct expression data in STRING (version 11.5), which were then transferred by homology to 148 of 336 HMP2 species. These findings highlight the abundance of coexpressed yet functionally uncharacterized proteins in gut metatranscriptomes.

On the basis of these initial assessments, we developed a method—FUGAsseM—to systematically predict functions of unknown proteins in the context of microbial communities (Fig. 1e). FUGAsseM is built on an integrative machine learning framework directly analogous to those used for single-organism function prediction tasks. It integrates multiple types of community-based data such as coexpression patterns from MTX, proximity in metagenomic assemblies (that is, genes occurring near each other in a contig), sequence similarity of proteins (that is, modeled coarsely as comembership in the same UniRef50 cluster^58^) and predicted domain–domain interactions^59^ (Methods). For each function of interest, FUGAsseM first builds an individual RF classifier for each data type in the first layer, which has the effect of mapping individual assay measurement values to its confidence level for functional relatedness, resulting in a prediction score indicating the likelihood of a protein family’s functional annotation based on its specific feature set. Next, it builds an ensemble classifier that combines RF predictions from all data types to provide final confidence of each protein family for a given function (Extended Data Fig. 1h). This process evaluates the relative contribution of each data type in predicting functional associations per gene per function, determining their overall informativeness in assigning functional annotations. FUGAsseM is designed for flexible annotation of gene sets from any source, as long as sufficient training data are available, making it broadly applicable across diverse functional categories such as GO terms^51^, Kyoto Encyclopedia of Genes and Genomes (KEGG) pathways^60^ or MetaCyc modules^61^. In this study, we focused on GO annotations as a proof-of-concept application, leveraging the extensive resources available for GO to demonstrate FUGAsseM’s predictive performance.

### FUGAsseM accurately predicts functions of uncharacterized proteins from microbial communities

**Fig. 2:**
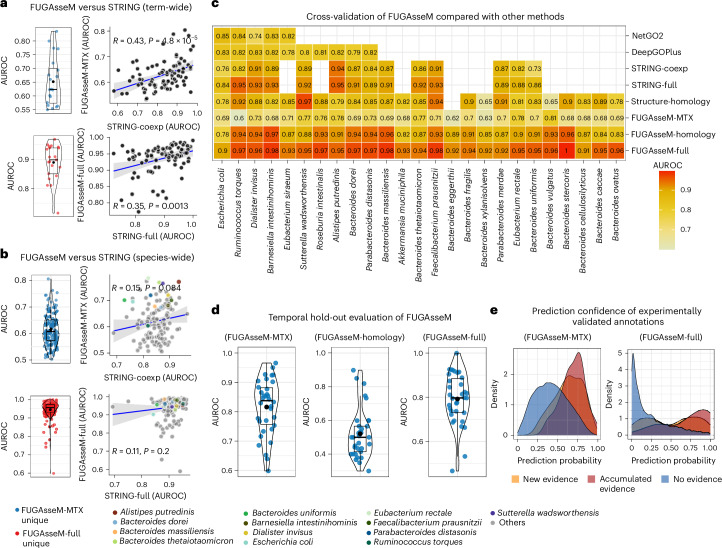
FUGAsseM predicts BP terms of microbial communities with high accuracy. **a**, FUGAsseM’s term-level performance strongly correlates with STRING’s isolate-based predictions and expands coverage to species lacking isolate data (*n* = 21 total terms). The Pearson correlation coefficients (95% confidence interval (CI)) and unadjusted *P* values between each pair of measurements are shown (*n* = 84 total terms). AUROCs were averaged per term per species (details in Supplementary Tables 5 and 6). Box plots display the median (line at the 50th percentile), IQR (box spanning the 25th to 75th percentiles), whiskers (extending to 1.5 × the IQR) and mean values (dark points). **b**, Across species, FUGAsseM shows comparable performance to STRING but supports more species (*n* = 147 total species). The Pearson correlation coefficients (95% CI) and unadjusted *P* values between each pair of measurements are shown (*n* = 140 total species). The full list is provided in Supplementary Tables 7 and 8. Box plots are displayed as in **a**. **c**, FUGAsseM matches the accuracy of state-of-the-art methods designed for single organisms while scaling to more species. AUROCs were averaged across the most abundant HMP2 species. Because of method limitations, only the top ten and five species were tested with DeepGOPlus and NetGO2.0, respectively. Only 14 of the top 25 species had isolate-based data in STRING (Supplementary Table 9). **d**, FUGAsseM-MTX and FUGAsseM-full models retained strong accuracy in predicting BP terms supported by newly accumulated experimental evidence (*n* = 34 total terms for temporal hold-out evaluation; Supplementary Table 10). Box plots are displayed as in **a**. **e**, FUGAsseM predicted significantly higher scores (GSEA method; FDR-adjusted *P* < 0.002 for multiple comparisons) for the annotations that lacked experimental evidence at *T*_0_ but gained accumulated experimental validation from *T*_0_ to *T*_1_ (that is, accumulated evidence) or totally unseen annotations at *T*_0_ with accumulated experimental validation at *T*_1_ (that is, new evidence). Source data

**Extended Data Fig. 2:**
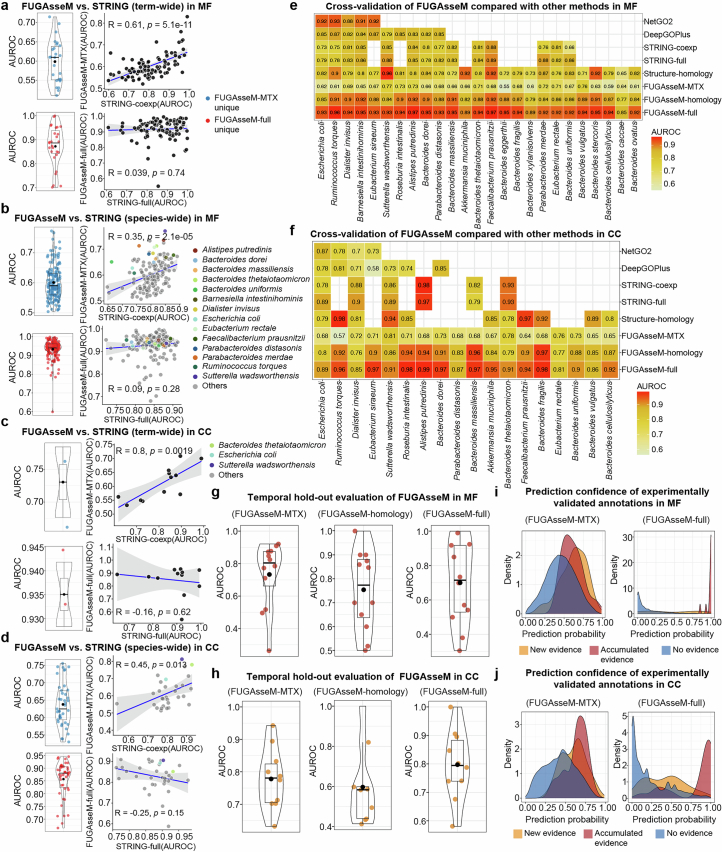
FUGAsseM accurately predicts MF and CC terms in microbial communities. (**a-d**) FUGAsseM predictions significantly correlated with STRING-based results (MF: *n* = 95 terms, 148 species; CC: n = 12 terms, 35 species) and identified additional terms in species lacking isolates (MF: n = 27 terms, 159 species; CC: *n* = 2 terms, 46 species). Pearson correlation coefficients (95% CI) and unadjusted *P* values shown. (**e,f**) FUGAsseM achieved comparable accuracy to DeepGOPlus and NetGO2.0; STRING data were available for 14 of 25 (MF) and 6 of 18 (CC) abundant species. (**g,h**) Hold-out validation confirmed high AUROC (MF: *n* = 12 terms; CC: *n* = 10 terms). (**i,j**) FUGAsseM predicted significantly higher scores (GSEA method; FDR-adjusted *P* < 0.002 for multiple comparisons) for annotations gaining experimental support over time. Box plots display the median (line at the 50th percentile), interquartile range (box spanning the 25th to 75th percentiles), whiskers (extending to 1.5× IQR), and mean values (dark points). Source data

To assess FUGAsseM’s prediction accuracy, we first compared its community-based predictions to those based on existing isolate data using a cross-validation approach. To this end, we trained FUGAsseM using isolate-based network data (that is, coexpression and integrated network data for isolates) from STRING (Methods). FUGAsseM’s performance using MTX coexpression alone (FUGAsseM-MTX) was comparable to STRING’s isolate coexpression (Fig. 2a, Extended Data Fig. 2a,c and Supplementary Table 5), with the added advantage of much broader applicability to any organism detected in a community (Pearson’s correlation for BP terms: *R* = 0.43, *P* < 0.0001). FUGAsseM’s performance was further improved by adding genomic proximity within metagenomically assembled contigs, sequence similarity and domain–domain interactions (that is, FUGAsseM-full), which also significantly correlated with predictions from STRING’s integrated data (Fig. 2a, Extended Data Fig. 2a,c and Supplementary Table 6). This also held true for species-wise comparisons across diverse GO terms but, again, applicable to any organism detected metaomically (Fig. 2b, Extended Data Fig. 2b,d and Supplementary Tables 7 and 8). Thus, FUGAsseM performed comparably to state-of-the-art data integration approaches but with the ability to infer functions for many more species directly from whole-community profiles.

**Extended Data Fig. 3:**
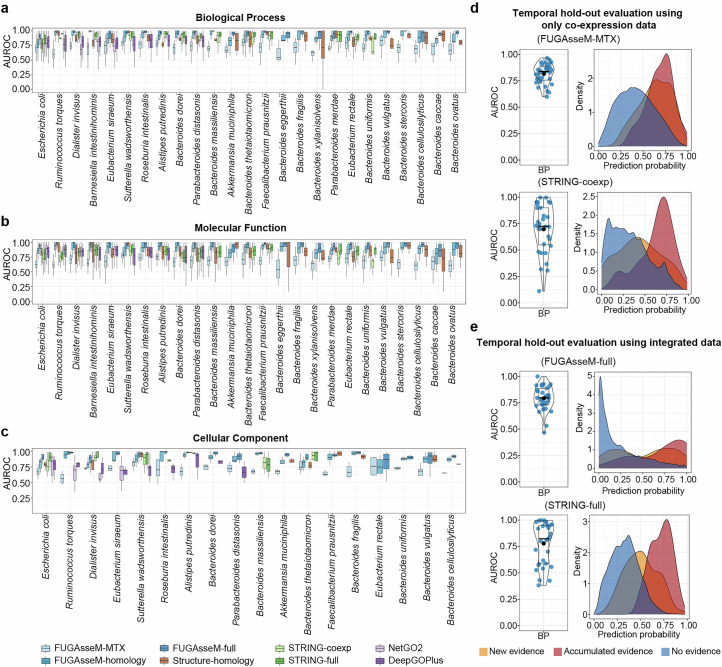
FUGAsseM achieves high accuracy and generalizability for GO term prediction. (**a-c**) FUGAsseM achieved AUROCs comparable to state-of-the-art methods across GO categories: BP (*n* = 1,010 term-species pairs), MF (*n* = 1,282 term-species pairs), and CC (*n* = 92 term-species pairs). (**d-e**) When predicting BP annotations with newly accumulated experimental evidence, FUGAsseM-MTX (**d**) and FUGAsseM-full (**e**) maintain high accuracy with community-wide data compared to those with isolate-based data (*n* = 34 total terms for temporal hold-out evaluation). ‘Accumulated evidence’: the annotations that lacked experimental evidence at T_0_ but gained accumulated experimental validation from T_0_ to T_1_; ‘New evidence’: totally unseen annotations at T_0_ with accumulated experimental validation at T_1_. Box plots display the median (line at the 50th percentile), interquartile range (box spanning the 25th to 75th percentiles), whiskers (extending to 1.5× IQR), and mean values (dark points). Source data

FUGAsseM’s accuracy was also comparable to that of other state-of-the-art methods for function prediction in single organisms (Fig. 2c). Two approaches that use sequence information with outstanding performance in CAFA3 (ref. ^22^) were selected for benchmarking: NetGO2.0 (ref. ^62^) and DeepGOPlus^63^. To perform the comparison, we applied DeepGOPlus with default parameter settings to the top ten most abundant species among those processed by FUGAsseM and NetGO2.0 to five species (a limitation of its web-based interface; Methods). FUGAsseM accurately predicted existing annotations with comparable performance to NetGO2.0, DeepGOPlus and STRING (Fig. 2c, Extended Data Fig. 3a and Supplementary Table 9). FUGAsseM-MTX achieved an average area under the receiver operating characteristic curve (AUROC) of 0.71 for informative BP term prediction. Strikingly, its AUROC was improved to 0.95 by aggregating other community-wide data (that is, FUGAsseM-full). Additionally, given the great advances in the field’s capacity for protein structure prediction^64–67^, we compared FUGAsseM to recent approaches based on structure homology when available (Methods). The results showed that FUGAsseM-MTX was comparable to predictions based on structural homology (while using only MTX covariation) and FUGAsseM-full still greatly outperformed both methods (Fig. 2c). This was one of the first demonstrations of synergy between multiple data types and multiple organisms within a community, also evidenced in additional evaluations below (Fig. 2c). Notably, FUGAsseM also remained accurate when predicting MF and CC terms (Extended Data Figs. 2e,f and 3b,c and Supplementary Table 9).

FUGAsseM further maintained its accuracy when predicting completely new annotations, using a temporally held-out annotation set analogous to that in CAFA’s evaluations^20–22^ (Fig. 2d,e). Methods relying on homology for prediction may be susceptible to circularity-induced overperformance (for example, protein *X* from gold standard is annotated to term *Y* because of homology to protein *Z*, while predictor also assigns *X* to *Y* because of homology to *Z*). Thus, we designed a temporally held-out evaluation inspired by CAFA, using UniProt^56^ annotations of HMP2 proteins lacking experimental evidence at the first time point (*T*_0_: release 2019_01, used by FUGAsseM for training) and adding experimental evidence by the second time point (*T*_1_: release 2022_01) (Methods). We trained FUGAsseM using HMP2 SC annotations available at *T*_0_, corresponding with GO in UniProt (release 2019_01). We then assessed its accuracy in predicting new annotations that were experimentally verified between then and *T*_1_ (release 2022_01), including ‘accumulated evidence‘ that gained new experimental validation at *T*_1_ and ‘new evidence’ that was unseen at *T*_0_ and newly added to the database with experimental validation at *T*_1_.

The only organism with sufficiently many new protein annotations proved to be *E.* *coli*; however, for this taxon, FUGAsseM’s MTX model and full model achieved high performance with an averaged AUROC of 0.80 (Fig. 2d, Extended Data Fig. 2g,h and Supplementary Table 10). The enrichment of newly experimentally validated annotations among high-confidence predictions was also highly statistically significant (gene set enrichment analysis (GSEA), *q* < 0.002; Fig. 2e and Extended Data Fig. 2i,j). Thus, FUGAsseM continues to perform well even when assessed using temporally held-out, newly experimentally validated function annotations. Similarly, we assessed the performance of FUGAsseM with STRING-based isolate data using this temporal hold-out approach (Methods). Strikingly, the FUGAsseM-MTX model outperformed STRING’s isolate-based coexpression for BP predictions (Extended Data Fig. 3d). Meanwhile, the FUGAsseM-full model closely matched STRING’s integrated predictions while excelling in identifying novel annotations with experimental support absent from the training data (Extended Data Fig. 3e).

**Extended Data Fig. 4:**
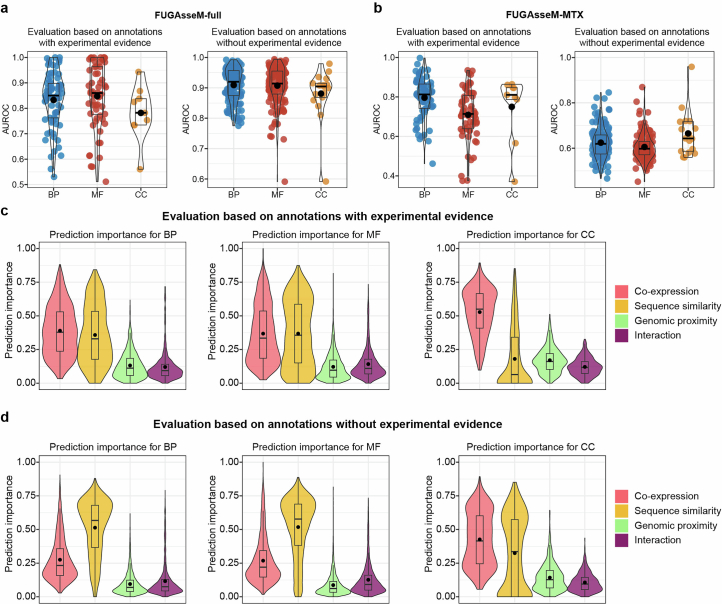
FUGAsseM accurately predicts annotations validated by experimental evidence. (**a,b**) The FUGAsseM-full (**a**) and FUGAsseM-MTX (**b**) models retained high accuracy when predicting annotations for experimentally validated annotations from the gold standard dataset (*n* = 142 and 285 total terms evaluated with and without experimental evidence, respectively). Box plots display the median (line at the 50th percentile), interquartile range (box spanning the 25th to 75th percentiles), whiskers (extending to 1.5× IQR), and mean values (dark points). (**c,d**) Distribution of Random Forest importance scores of FUGAsseM-full model’s second (data integration) layer, which predicted annotations with (**c**) (*n* = 3,725 term-species pairs for prediction) and without (**d**) (*n* = 11,513 term-species pairs for prediction) experimental time in the gold standard set. Box plots as in **a**. Source data

To further assess the robustness of FUGAsseM and minimize confounding from the potential circularity of homology-based annotations, we evaluated its performance using only experimentally confirmed annotations from our gold-standard set (Methods). The FUGAsseM-full model consistently demonstrated high predictive accuracy across both experimentally validated and other annotations, indicating its effectiveness in mitigating these potential biases (Extended Data Fig. 4a). Furthermore, the FUGAsseM-MTX model exhibited much higher performance for experimentally validated annotations compared to those lacking experimental support, reinforcing the reliability of MTX-derived functional predictions (Extended Data Fig. 4b). This expanded analysis confirms that FUGAsseM’s performance advantages persist independently of homology effects.

### MTX-based coexpression contributes substantially to FUGAsseM predictions

**Fig. 3:**
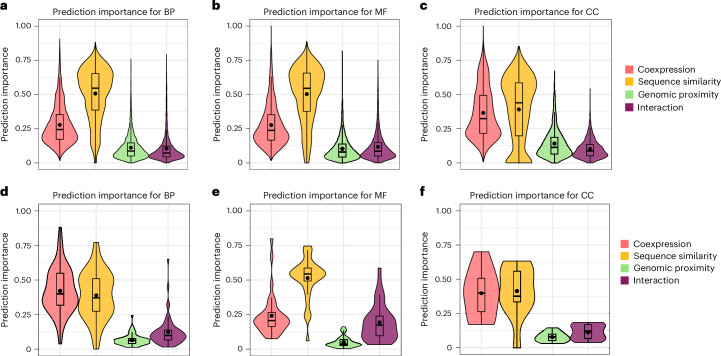
MTX-based coexpression contributes substantially to FUGAsseM-full protein function predictions. **a**–**c**, Distribution of RF importance scores from the FUGAsseM-full model’s second (data integration) layer. Only GO terms with sufficient performance (resulting in predictions with confidence probability ≥ 0.75) are included for BP (**a**; *n* = 14,249 total term–species pairs for prediction), MF (**b**; *n* = 18,553 total term–species pairs for prediction) and CC (**c**; *n* = 1,623 total term–species pairs for prediction). The full list is provided in Supplementary Table 11. **d**–**f**, Distribution of importance scores for successful FUGAsseM models (those that assigned GO annotations to proteins with a prediction probability ≥ 0.75) showing newly predicted annotations that were not used for training, based on accumulated experimental evidence over time (*n* = 65 total term–species pairs for BP (**d**), 34 total term–species pairs for MF (**e**) and 11 total term–species pairs for CC (**f**)). The full list is provided in Supplementary Table 12. Box plots display the median (line at the 50th percentile), IQR (box spanning the 25th to 75th percentiles), whiskers (extending to 1.5 × the IQR) and mean values (dark points). Source data

We further explored the contributional importance of each data type integrated in the FUGAsseM-full model. To this end, we assessed the importance scores learned by the second-layer RF for accurate GO terms (that is, successful models), where the latter were defined as the models that resulted in high-confidence (that is, prediction probability ≥ 0.75) predictions of GO annotations. Both MTX-based coexpression and sequence similarity achieved average importance scores over 0.28, exceeding that of the third most important data type (genomic proximity, 0.11) (Fig. 3a–c and Supplementary Table 11). Their importance was also much higher than proximity (proteins assembled in the same contig) and predicted domain–domain interactions, highlighting the important role of MTX-based coexpression in function prediction.

MTX-based coexpression maintained a large contribution to predicting annotations with new experimental evidence over time, drawing from the corresponding evaluation above (Fig. 3d–f and Supplementary Table 12). To avoid potential circularity for predicting annotations that were made previously on the basis of the same type of evidence, we examined the importance of data types for predicting new annotations excluded from the FUGAsseM’s training models. Strikingly, MTX-based coexpression achieved a notable contribution (average importance scores ≥ 0.42) to predicting new BP annotations even when these annotations were made using other data types (for example, sequence similarity of 0.39) (Fig. 3d). Given that MF terms are often carried out by the action of a single macromolecule, sequence similarity remained a high contributor to predictions of MF terms (Fig. 3e), consistent with previous findings^21^. This provides an interesting demonstration that MTX coexpression can be informative for function assignment in a manner directly analogous to that of transcriptional coexpression within single organisms under controlled conditions^36^.

Moreover, we conducted additional analyses using experimentally validated annotations from our gold-standard set, effectively reducing potential confounding from homology-based annotation transfers (Methods). Notably, MTX-based coexpression features contributed more prominently to predicting annotations with experimental validation in the integrated model compared to other types of data (Extended Data Fig. 4c,d). This suggests that incorporating MTX-derived coexpression features enhances functional inference, reinforcing their value in improving annotation accuracy and reliability.

**Extended Data Fig. 5:**
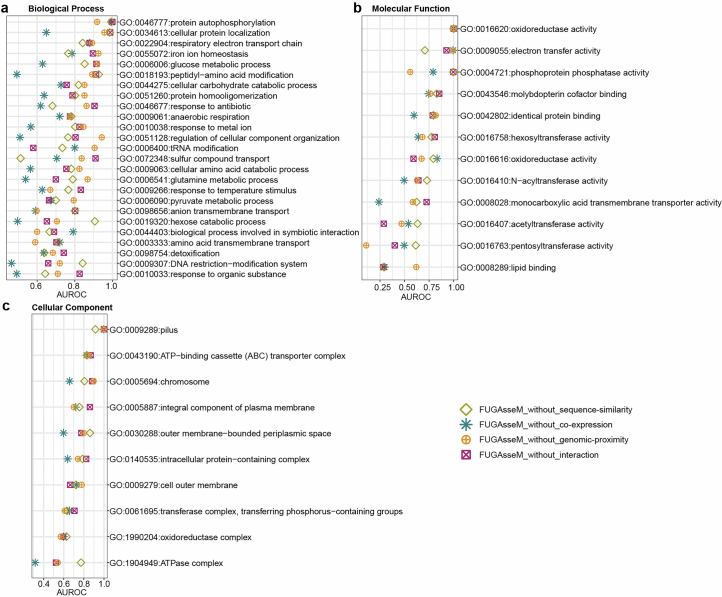
Temporal hold-out evaluation by removing one data type at a time. Removing MTX-based coexpression showed dominant influence on the performance of FUGAsseM to predict new annotations that accumulated experimental evidence over time for (**a**) Biological Processes, (**b**) Molecular Functions, and (**c**) Cellular Components. Averaged AUROC of each term was calculated across the HMP2 species used in this study. The top 25 GO terms with the highest average AUROC across species and methods are shown (or all available terms if fewer than 25 are present). Source data

Furthermore, we performed an ablation study to quantify the contribution of each evidence type to the prediction of FUGAsseM-full models, systematically training FUGAsseM models while excluding one data type at a time (Methods). To minimize potential homology-based circularity, we used a temporal hold-out validation approach, ensuring that predictions were derived from independent multiomics evidence rather than propagated database annotations. This analysis revealed that MTX-based coexpression remained a key predictive feature across all GO aspects, with particularly strong contributions to BP annotations (Extended Data Fig. 5). These findings underscore the critical role of MTX-derived signals in capturing functional relationships within microbial communities.

### Predicted high-confidence functional annotations refine the functional landscape of the human microbiome

**Extended Data Fig. 6:**
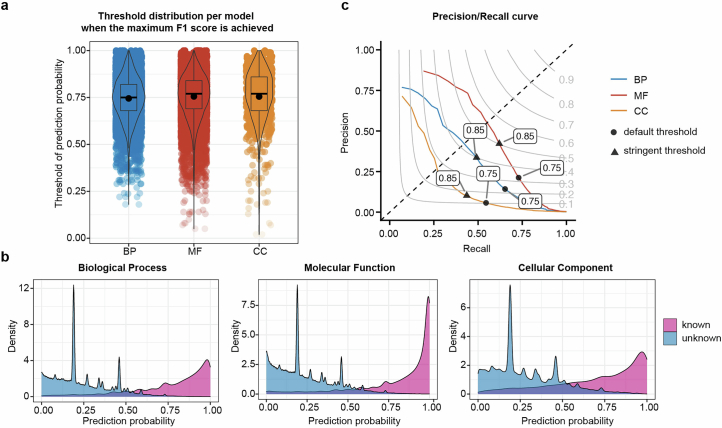
Threshold selection for high-confidence predictions of FUGAsseM. (**a**) The thresholds of prediction confidence when achieving the maximum F1 score were heterozygous across models (here, Random Forest models) used for predicting each term per species (*n* = 21,785 total term-species pairs for prediction). Box plots display the median (line at the 50th percentile), interquartile range (box spanning the 25th to 75th percentiles), whiskers (extending to 1.5× IQR), and mean values (dark points). (**b**) In addition, known annotations in UniProt tended to be predicted with higher confidence than those unknowns in all types of GO aspects. A threshold like 0.75 prediction probability looks strict enough to cover most of known annotations with high confidence. (**c**) Though the threshold of 0.75 achieved high recall while keeping new potentially true predictions, it still looks ‘default’ with low precision. We defined a ‘stringent’ threshold (that is, 0.85 prediction probability) that doubled the precision while maintaining recall. However, a more ‘stringent’ threshold makes it more possible to miss true new predictions meanwhile. Source data

Because the preceding evaluations provided high confidence in FUGAsseM’s accuracy, we next applied it to predict community-encoded functions (that is, informative GO annotations) of the human gut microbiome. We integrated coexpression patterns from 800 HMP2 metatranscriptomes and proximity patterns from assemblies of 1,595 HMP2 metagenomes, computed sequence similarities modeled coarsely as comembership within UniRef50 and drew domain–domain interactions from our previous published results^7^ for the HMP2 protein families (Methods). To determine whether a target protein is confidently assigned to a function, we defined a cutoff for new predictions with high confidence. These were optimized per term and taxon by maximizing expected *F*_1_ score (Extended Data Fig. 6a). Predictions with known annotations in UniProt tended to achieve higher confidence than those without (Extended Data Fig. 6b), acting as positive controls. Given these, we used prediction probability 0.75 as a uniform threshold for all models to define high-confidence predictions (termed the ‘default’ threshold) (Extended Data Fig. 6c). This retained true positives with high prediction confidence while including new predictions. Next, we defined prediction probability 0.85 as a ‘stringent’ threshold for higher precision, maintaining a sufficient true positive rate but at an especially low false positive rate.

**Extended Data Fig. 7:**
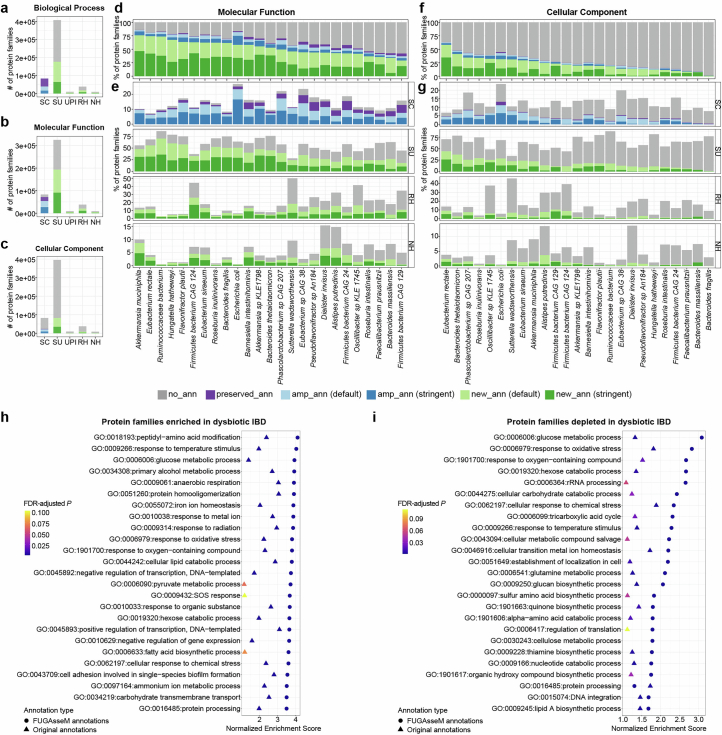
Functional repertoire of the gut microbial protein families is expanded by FUGAsseM in the HMP2. (**a-c**) FUGAsseM assigned high-confidence annotations to previously uncharacterized protein families across all GO aspects—BP (**a**), MF (**b**), and CC (**c**)—in the HMP2. (**d–g**) Among the top 25 most uncharacterized HMP2 species with the greatest number of novel proteins, many novel proteins were annotated with high-confidence MF and CC terms, improving functional coverage for both well-studied and poorly characterized taxa. Predictions were defined at two thresholds: ‘default’ (probability ≥ 0.75) and ‘stringent’ (≥ 0.85). Categories include: ‘no_ann’ (no high-confidence predictions), ‘preserved_ann’ (proteins already annotated in UniProt), ‘amp_ann (default/stringent)’ (known proteins with new predictions), and ‘new_ann (default/stringent)’ (uncharacterized proteins with new annotations). Full results are in Supplementary Table 13. (**h-i**) FUGAsseM annotations also captured biologically relevant signals associated with inflammatory bowel disease (IBD). Gene Set Enrichment Analysis (GSEA) showed that proteins prioritized by MetaWIBELE in IBD were more strongly enriched in FUGAsseM-predicted annotations. Normalized enrichment scores (NES) revealed significant enrichment (**h**) and depletion (**i**) among protein families associated with IBD. Here, NES is an adjusted enrichment score by correcting differences of gene-set sizes, reflecting the degree to which a gene list is overrepresented at the top of a gene list ranked by MetaWIBELE’s prioritization. The top 25 BP most significantly enriched terms (GSEA test; FDR-adjusted *P* < 0.25 for multiple comparisons) are listed in decreasing order by NES. Source data

Among the 546,251 protein families from the HMP2 species processed by FUGAsseM, we assigned high-confidence (with the default cutoff) predictions to 443,549 families, including 267,944 protein families with GO BP annotations (that is, 49.1% of the total), 364,652 with MF annotations (that is, 66.8% of the total) and 120,134 with CC annotations (that is, 22.0% of the total) (Extended Data Fig. 7a–c). In total, 364,965 of these were previously uncharacterized (that is, 82.3% of all predictions), including 33,912 novel protein families (that is, RH and NH with homology at <90% identify or 80% coverage to any proteins in UniProt; 20,456 predicted with BP annotations, 24,438 predicted with MF annotations and 9,608 predicted with CC annotations) with comparable prediction confidence to previously annotated proteins. Notably, quantitative analysis showed that FUGAsseM substantially captured functions that were also significantly enriched among protein families previously prioritized as bioactive in IBD^7^ (Extended Data Fig. 7h,i). This result showcases FUGAsseM’s ability to assign putative functions to uncharacterized yet phenotypically important proteins in IBD.

**Fig. 4:**
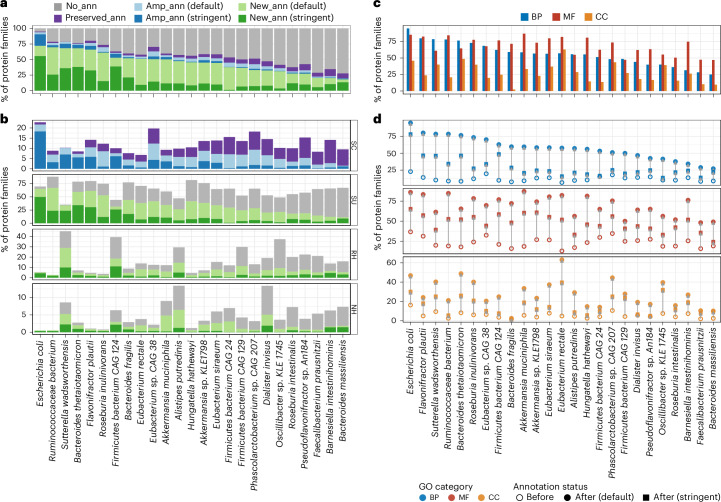
FUGAsseM predictions greatly expand putative function assignments in the human gut microbial gene catalog. **a**, High-confidence BP annotations were assigned to the top 25 most uncharacterized HMP2 species (that is, those encoding the greatest numbers of novel proteins). These species from the community showed different levels of functional characterization, including sometimes expanding the community pangenomes of species with otherwise well-characterized strains (for example, *E.* *coli*). Here, default (prediction probability 0.75) and stringent (0.85) thresholds are used to define high-confidence predictions. ‘No_ann’, protein families that were not assigned any high-confidence predictions; ‘Preserved_ann’, characterized protein families annotated in UniProt; ‘Amp_ann (default)’, characterized proteins assigned new predictions at the default threshold; ‘Amp_ann (stringent)’, the same for the stringent threshold; ‘New_ann (default)’, uncharacterized protein families assigned one or more new predictions using the default threshold; ‘New_ann (stringent)’, the same for the stringent threshold. **b**, Predicted functions not only expanded the function capacity of well-studied species but also improved the characterization of less studied species in the human microbiome. Both characterized proteins and uncharacterized proteins had better functional annotations. The full list is provided in Supplementary Table 13. **c**,**d**, Moreover, these predicted functions spanned all three aspects of GO (**c**) and benefited from data integration within FUGAsseM, taking advantage of different data types (**d**). The full list is provided in Supplementary Table 14. ‘Before’, not processed by FUGAsseM; ‘After (default)’, processed with the default threshold; ‘After (stringent)’, processed with the stringent threshold. Source data

High-confidence predicted annotations expanded our understanding of microbial protein functions from both well-studied and less studied species in the human gut (Fig. 4 and Extended Data Fig. 7d–g). Focusing on the top 25 species with the greatest number of novel (that is, NH and RH) proteins, on average, the fraction of protein families annotated with BP terms per taxon expanded from 12.1% to 57.4% (4.7-fold increase) on the basis of the default threshold and to 28.8% (2.4-fold increase) on the basis of the stringent threshold (Fig. 4a). Most of these predictions were for the pangenomes of common gut taxa such as *Bacteroides thetaiotaomicron*, *Ruminococcaceae bacterium* and *Roseburia inulinivorans*. This emphasizes the degree to which even species with well-characterized strains (for example, *E.* *coli*, which is among the top 25 species) may still have undercharacterized community pangenomes (Extended Data Fig. 1b); in such cases, FUGAsseM strikingly improved pangenome characterization (Fig. 4b and Supplementary Table 13). Other less studied species such as *Sutterella wadsworthensis* and *Firmicutes bacterium CAG 124* with many novel proteins were also much better annotated (with an average of 25.6% of novel proteins annotated using stringent threshold). Moreover, 9,784 (of 15,638) protein families previously annotated with informative GO annotations were assigned to more informative GO terms, expanding their characterization (Fig. 4b and Extended Data Fig. 7e,g). In total, 62,209 (of 116,740) uncharacterized protein families (that is, lacking informative GO annotations) were assigned high-confidence functional annotations. Overall, gut microbial proteins gained extensive high-confidence predictions across GO aspects, expanding the functional repertoire of the gut microbiome.

To expand statistics for functional annotations spanning all of GO’s aspects, we observed a 3.4-fold increase in BP annotations (mean ± SD: 11 ± 29 annotations per term per species in the training set versus 37 ± 136 in the high-confidence prediction set), a 4.3-fold increase in MF annotations (10 ± 25 versus 43 ± 129) and a 4.1-fold increase in CC annotations (21 ± 41 versus 86 ± 190) on the basis of the default confidence threshold (Fig. 4c). In particular, annotations for BP terms with high confidence were substantially increased by both the default and the stringent thresholds (Fig. 4d and Supplementary Table 14). Most of these predictions benefited from the contribution of MTX-based coexpression (Fig. 3a,d). Conversely, as is typical for many of their terms, newly predicted MF and CC annotations sometimes represented general functions that require additional knowledge of the context in which the gene product acts. They were also more likely to benefit from the contribution of sequence similarity for prediction (Fig. 3). Our analysis reveals a substantial number of microbial proteins in the human gut that remain uncharacterized. While their precise functional roles are yet to be fully elucidated, their presence in transcriptionally active communities suggests potential biological importance.

### Uncharacterized proteins increase the diversity of species-shared and species-specific functions in the human gut

**Fig. 5:**
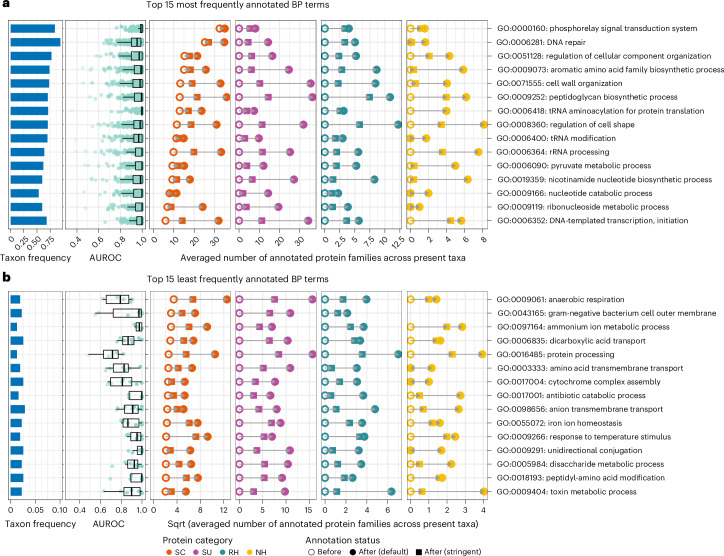
Uncharacterized proteins are predicted to be broadly distributed and species-specific functions with high confidence in the human microbiome. **a**, Enumeration of the fraction of annotated taxa (first column), AUROC values per taxon (second column; *n* = 3,297 total term–species pairs for prediction) and numbers of annotations (third column) for species-shared BP terms predicted by FUGAsseM in the HMP2. The top 15 terms with the largest number of species with at least one assignment are listed in decreasing order of average preserved annotations across species before running FUGAsseM (full results in Supplementary Tables 15 and 16). Box plots display the median (line at the 50th percentile), IQR (box spanning the 25th to 75th percentiles) and whiskers (extending to 1.5 × the IQR). **b**, Fraction of annotated taxa (first column), AUROC values per taxon (second column; *n* = 100 total term–species pairs for prediction) and numbers of annotations (third column) for species-specific BP terms predicted by FUGAsseM from the HMP2 cohort. The 15 least frequently predicted terms are listed in decreasing order of mean number of preserved annotations as in **a** (full results in Supplementary Tables 17 and 18). Box plots are displayed as in **a**. Source data

The top 15 most frequently predicted BP terms were assigned to more than half of the total species in the HMP2 by FUGAsseM, furthering their characterization (Fig. 5a, first column, and Supplementary Tables 15 and 16). To select the most frequently predicted terms in HMP2, we sorted all predicted terms in descending order on the basis of the count of species that were assigned at least one protein to each corresponding term. Most of these functions were related to essential or housekeeping activities (for example, DNA processing, RNA processing, nucleotide or amino acid biosynthetic process and CC organization). FUGAsseM achieved an average cross-validated AUROC of more than 0.95 over these top 15 terms (second column in Fig. 5a), indicating the high confidence of these predictions. The increase in the annotations of uncharacterized protein families (average of 33.84 and 12.06 protein families per term per species annotated with default and stringent thresholds, respectively) suggests undercharacterized diversity of microbial proteins for these essential functions (Fig. 5a, third column). For example, 542 novel proteins from 48 species were newly predicted to be involved in the regulation of cell shape (GO:0008360), greatly increasing the protein diversity of this process.

FUGAsseM also improved the diversity of species-specific functions (Fig. 5b and Supplementary Tables 17 and 18). We examined the top 15 least frequently predicted BP terms that were the bottom 15 terms from the ranking list (introduced above). These terms were often specific to a few species but with good performance (average AUROC of 0.88). Unlike the housekeeping processes shared by many species, these functions were enriched for highly specific metabolic processes, including toxin metabolism (for example, altering the toxicity of ingested compounds and impacting health outcomes^68^), iron ion homeostasis (for example, changing iron availability impacting on host-microbiota interactions^69,70^) and ammonium ion metabolism (for example, affecting enteric nervous system function^71^). Many nonmetabolic processes were also predicted with high confidence and were often related to interaction between microorganisms and their host or environment. These included outer-membrane assembly, which is responsible for binding of and response to host proteins^72,73^, transmembrane transport, which has a role in nutrient uptake^74,75^, and dicarboxylic acid transport, which is involved in the uptake of succinate, fumarate and malate by the microbiome^76^. Species-specific functions might be underestimated previously as housekeeping functions, which are typically easier to predict, dominate the annotations. The increased diversity of such specialized functions by uncharacterized proteins (including novel proteins) suggests richness of unexplored functions, which potentially distinguish clades and contribute to the fitness of both microorganisms and the host.

### Newly annotated proteins in microbial nutrient use and phage defense

**Fig. 6:**
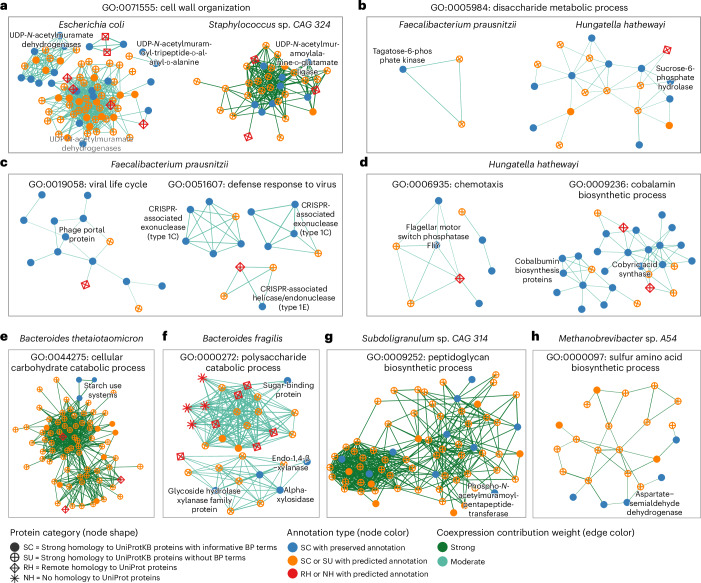
Selected examples of specific newly predicted function annotations spanning pathways and functions in the human gut microbiome. **a**–**h**, Examples of newly predicted high-confidence annotations ranged across housekeeping process (**a**), species-specific functions (**b**), common gut taxa (**c**,**d**) and less studied species with numerous novel proteins (**e**–**h**), demonstrating strong correlation between transcripts of uncharacterized protein families (that is, SU, RH and NH) and characterized families (that is, SC) for diverse BP terms in the HMP2. Each node represents a protein family that was assigned to the corresponding term from preserved annotations or by FUGAsseM’s prediction with the stringent threshold. Nodes are linked when the correlation coefficient of the transcripts exceeds the 90th percentile of all MTX-based correlations per species per term. The width of each edge represents the correlation coefficient. Edges are colored on the basis of the prediction importance of MTX-based coexpression in FUGAsseM’s integrated models, where coexpression importance < 0.1 represents a ‘weak’ contribution, importance > 0.5 represents a ‘strong’ contribution and other edges represent a ‘moderate’ contribution from coexpression. Source data

A wide array of taxa and functions were covered by new FUGAsseM predictions in the human gut, ranging from broad housekeeping processes to highly taxon-specific nutrient use and phage responses (Fig. 6). For example, the annotations of housekeeping functions (for example, cell wall organization GO:0071555)^77^ were greatly expanded in multiple well-studied and less studied species (Fig. 6a, Supplementary Table 19 and Supplementary Note 1). Similarly, species-specific annotations such as disaccharide metabolism (GO:0005984) were also improved (Fig. 6b and Supplementary Table 20). Many bacteria in the human gut use disaccharides as an important energy source^78^ and these were better characterized in health-relevant microorganisms such as *Faecalibacterium prausnitzii* and *Hungatella hathewayi* (Supplementary Note 1). Lastly, the importance of coexpression for function prediction was also affirmed by these data, with many uncharacterized proteins receiving new annotations on the basis of strong coexpression with annotated proteins in their source species despite lacking annotated homologs (Fig. 6c,d and Supplementary Tables 21 and 22). For example, *H.* *hathewayi* is associated with colorectal cancer and has the ability to break down various glycosaminoglycans, aiding colonization^79^. The use of FUGAsseM resulted in the assignment of high-confidence functional annotations to 3,231 uncharacterized proteins of *H.* *hathewayi*, including chemotaxis (a fitness attribute in nutrient foraging^80^; GO:0006935) and cobalamin biosynthesis (contributing to glycosaminoglycan-degrading^79^; GO:0009236) (Fig. 6d and Supplementary Note 1).

**Extended Data Fig. 8:**
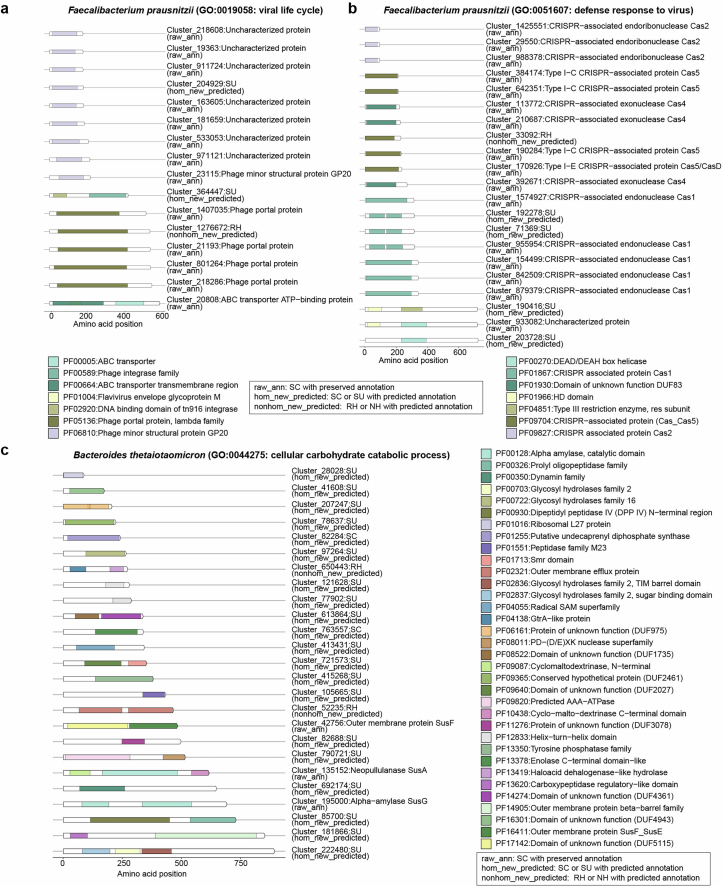
Domain architectures support FUGAsseM function predictions in *Faecalibacterium prausnitzii* and *Bacteroides thetaiotaomicron.* (**a**) Proteins predicted with viral life cycle (GO:0019058) showed similar domain architectures with the previously annotated proteins, clustering into two main phage clusters. (**b**) Similarly, proteins predicted with defend response to virus (GO:0051607) exhibited domain patterns characteristic of two distinct CRISPR systems. Proteins shown were also strongly coexpressed (Fig. 6c). (**c**) In *B. thetaiotaomicron*, proteins predicted with cellular carbohydrate catabolic process (GO:0044275) contained glycosyl hydrolase and outer membrane—key components of the starch utilization system for carbohydrate metabolism. Domain architectures of randomly selected 25 *B. thetaiotaomicron’*s proteins predicted with GO:0044275 (strongly coexpressed in Fig. 6e) are shown. Source data

*F.* *prausnitzii*, which has been reported to be associated with health^44^ and exhibits high phylogenetic diversity^7,81^, showed largely expanded characterization of its pangenomes, which are at present notably underannotated given its health relevance (Figs. 1b and 4). Strikingly, uncharacterized *F.* *prausnitzii* proteins confidently predicted as viral responsive (GO:0019058 and GO:0051607) were strongly coexpressed with a small number of previously characterized pathway members (Fig. 6c). Previous studies showed that *F.* *prausnitzii* phages were enriched in IBD^82^, including Myroviridae type I cluster 6 (Taranis and Toutatis) and Siphoviridae type I cluster 1 (Lugh) families. Although *F.* *prausnitzii* uncharacterized proteins lacked global sequence similarity to related previously annotated phage proteins (Extended Data Fig. 8a and Supplementary Table 21), their similar domain architectures and coexpression confirmed their functional relatedness. In addition, the subunits of types 1C and 1E, the most common CRISPR systems of *F.* *prausnitzii* strains^83^, were newly predicted in *F.* *prausnitzii*. These spanned Cas1 or Cas5 and Cas3 genes from type 1E and 1C systems, respectively (Extended Data Fig. 8b). These previously annotated and newly predicted CRISPR–Cas proteins showed 60–80% amino acid sequence identity and strong coexpression within system types and their similarity dropped to ~20% between system types with a weak correlation at the expression level. These findings emphasize the informative role of MTX-based coexpression in deducing functional capacity, complementing sequence similarity.

In another case, proteins involved in carbohydrate catabolism (GO:0044275) were newly predicted in *Bacteroides*, a dominant clade and also a primary glycan degrader in the human gut microbiota. This activity is mediated by polysaccharide use loci (PULs), which typically include multiple carbohydrate active enzymes (CAZymes) that enable complex carbohydrate recognition, uptake and breakdown^84–87^. Although the newly predicted proteins in *B.* *thetaiotaomicron* involved in GO:0044275 lacked significant homology to annotated PUL proteins, their domain architectures tended to be similar, including glycosyl hydrolase Pfam domains, outer-membrane-related domains and PUL domains from the same operon (Extended Data Fig. 8c). Again, MTX-based coexpression showed the strongest contribution to the predictions of carbohydrate catabolic processes in *B.* *thetaiotamicron* (Fig. 6e and Supplementary Table 23). Similarly, in *Bacteroides* *fragilis*, coexpression patterns revealed associations between these glycoside hydrolases and other known PUL proteins^88,89^, indicating the concerted action of protein sets (Fig. 6f, Supplementary Table 22 and Supplementary Note 1). These extended annotations shed light on the crucial role of *Bacteroides* in polysaccharide metabolism for energy harvesting, aiding in understanding both colonization and ecological persistence.

In addition to these examples in well-studied species, FUGAsseM provided insights into protein function for less studied species. In many less studied species, the MTX-based expression of novel proteins was strongly correlated with annotated pathway members, indicative of underlying functional associations active specifically within a community context (Fig. 6g,h). For example, uncharacterized proteins in *Subdoligranulum* spp., which have a role in carbohydrate fermentation^8,90^ and are associated with chronic-inflammation-related immune markers^91^, were predicted with high confidence by leveraging coexpression patterns. In particular, FUGAsseM expanded the repertoire of proteins contributing to peptidoglycan synthesis (for example, phospho-*N*-acetylmuramoyl-pentapeptide transferase) in *Subdoligranulum* (Fig. 6g and Supplementary Table 22), which is a key component in sensing by host proteins and contributing to the regulation of inflammatory response toward bacteria^92,93^. Additionally, uncharacterized proteins from undercharacterized species such as *Methanobrevibacter* spp. were predicted to be associated with amino acid biosynthesis enzymes (Fig. 6h and Supplementary Table 22), such as aspartate–semialdehyde dehydrogenase, which participates in sulfur amino acid processes unique to these Archaea and associated with metabolic disease^94,95^. Taken together, FUGAsseM’s coexpression-based function prediction furthered the characterization of both well-studied and less studied members of the gut microbiome.

## Discussion

While culture-independent sequencing has provided increasingly vast catalogs of microbial community gene families, a large majority of their functions remain unknown. Here, we present a new method, FUGAsseM, that provides precise predictions of functions for uncharacterized proteins by integrating diverse community-wide data, including MTX-based coexpression, MGX-based genomic proximity, sequence similarity and predicted domain–domain interactions. FUGAsseM uses a two-layered RF classifier system to assign proteins to new functions through guilt by association. For a given function, individual classifiers are initially trained for each data type to assign new proteins to that function on the basis of their associations with other annotated proteins. In the second layer, an ensemble classifier integrates the per-evidence prediction confidence scores from the first layer to produce a unified confidence score. This architecture allows for flexible adjustment of evidence weighting on a function-by-function basis, thus capturing biological trends that favor greater overall prediction accuracy. FUGAsseM can incorporate a large diversity of data types, while also scaling efficiently with the number of datasets per type. We used FUGAsseM to assign high-confidence GO annotations (that is, BP, MF and CC) to >443,000 protein families in the human gut, including >33,000 families with <90% identity or <80% coverage to any UniProt member. These predictions substantially expanded the functional repertoire of both common gut taxa (for example, *F.* *prausnitzii*) and less studied species (for example, *S.* *wadsworthensis*) and ranged from broadly distributed housekeeping activities to highly specialized metabolic functions. The functional diversity of uncharacterized proteins was particularly well informed by the analysis of MTX-based coexpression. An open-source implementation of FUGAsseM, supporting documentation, tutorials and all current data products (including function predictions of HMP2 protein families) are available online (http://huttenhower.sph.harvard.edu/fugassem).

FUGAsseM is modeled on computational strategies for protein function prediction in single organisms, which have become extremely accurate over the past two decades^17,18^. These, in turn, build on simple annotation transfer by homology, under the assumption that proteins with sufficiently similar sequences frequently carry out similar functions^23–26^. Particularly with the advent of deep-learning-based structural modeling, this intuition can be extended to include function transfer based on structural similarity^19,96,97^. A wide range of other data types have also been used for function prediction or transfer, including gene neighborhood methods (that is, relating genes located in the same genomic loci, such as operons^27,98,99^) and phylogenetic profiling (that is, relating genes on the basis of co-occurrence across species^97,100^). Network-based approaches rely on guilt by association for prediction, such as protein–protein interactions^101–103^, epistatic genetic interactions^104,105^ or coexpression^106^ from DNA microarrays^35,36,49^, qPCR^107^ or RNA-seq^50^.

**Extended Data Fig. 9:**
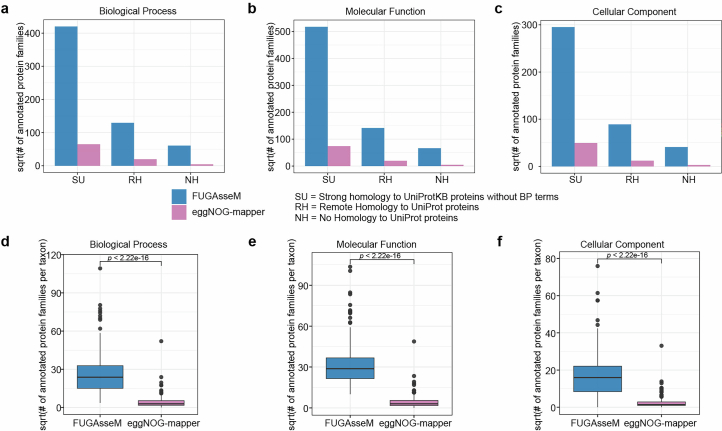
FUGAsseM predicted more protein families with informative GO annotations compared to eggNOG-mapper. (**a-c**) Total number of annotated protein families by FUGAsseM (full model with integrated data) and eggNOG-mapper, including proteins with and without homologs to UniProtKB proteins. Only predictions with high confidence (prediction probability ≥0.75 for FUGAsseM, e-value < 0.001 for eggNOG-mapper) are included for (**a**) Biological Processes, (**b**) Molecular Functions, and (**c**) Cellular Components. (**d**-**f**) Distribution of annotated protein families with high confidence per species are shown (*n* = 620 total method-species pairs). Statistical analysis was performed using two-sided unpaired Wilcoxon tests with unadjusted *P* values reported. Box plots display the median (line at the 50th percentile), interquartile range (box spanning the 25th to 75th percentiles), whiskers (extending to 1.5× IQR), and outliers (values beyond 1.5× IQR from the quartiles). Source data

However, analogous function prediction methods for microbial communities have not been previously developed. Compared to single organisms, microbial communities tend to contain organisms with substantially more diverse genetic backgrounds, thus introducing additional complexity for function prediction. Even human-associated communities typically contain a high proportion of novel sequences lacking homology to known proteins and, thus, with limited knowledge of genomic context, protein structure or protein–protein interaction. Approximately 45% of assembled protein families in the human microbiome were new (RH and NH with homology with <90% amino acid sequence identity or <80% coverage to any UniProt member) and that fraction was even larger in undercharacterized microbial communities such as marine or soil communities (~70% to 90%)^7^. Thus, community-wide function prediction requires a combination of leveraging community-wide data directly, transferring function among organisms using more than simple homology and scalable computational methods, as direct experimental characterization would be challenging. FUGAsseM, thus, represents one of the first efforts to specifically predict functions in complex microbial communities. It enhances the functional characterization of the human microbiome, with the percentage of protein families assigned to informative BP terms increasing from 14.4% to 49.1% (3.4-fold increase). By comparing the number of annotated families assigned by FUGAsseM to eggNOG-mapper^108^ (which assigns protein families by single-best-hit sequence homology), FUGAsseM clearly demonstrated superior coverage of meaningful annotations compared to eggNOG-mapper, particularly for proteins lacking notable homologs in UniProt (Extended Data Fig. 9 and Supplementary Table 24). This underscores the strength of the FUGAsseM framework in accurately predicting previously uncharacterized protein functions within microbial communities.

FUGAsseM differs from previous single-organism methods mainly by using community-level data designed specifically for the complexity of microbiomes. Single-organism algorithms developed for analyzing isolate genomes are limited in the context of communities^23–25,62,63^. These tools typically rely on the existence of functional data (gene expression, protein interactions, etc.) for microbial isolates^57,106^ that do not exist for the vast majority of microbiome members. For example, only two of 336 HMP2 species have direct expression data in STRING. In their absence, functions are often transferred on the basis of homology, which is highly variable and unreliable without additional supporting data. FUGAsseM addresses these limitations by incorporating advanced multiomics techniques, allowing a much larger number of sequences from communities to receive at least putative functional interpretations. The combination of MGX with MTX makes numerous protein sequences from communities accessible, allowing for a more comprehensive exploration of microbial functionality in ecosystems. By applying FUGAsseM’s integrative prediction approach to whole-community data (for example, MTX-based coexpression, MGX-based genomic proximity and across-species sequence similarity), we can address proteins with little homology and for which no isolate functional data are available.

MTX coexpression for function prediction in communities is of particular note, as it may be argued that coexpression analysis from microarrays unlocked the annotation potential of genomes as they began to be sequenced in the early 2000s^35^. Unlike an isolate’s expression profiles from a monoculture environment—which are already highly informative^57^—MTX captures the transcriptional profiles of microorganisms in situ^40,41,109,110^. It also has the potential to cover the same set of taxa and genes spanning communities as profiled by typical metagenomic DNA sequencing methods. Particularly in combination with other community-relevant data, such as cross-species homology, FUGAsseM is especially suitable even for communities without isolates. Moreover, FUGAsseM’s MTX-based approach can reduce the impact of circularity in function prediction (for example, training sequence similarity to predict functions that are themselves annotated through sequence similarity). Correspondingly, our evaluations on predictions based on experimental evidence (independent from the training set) showed substantial contribution from MTX-based coexpression to high-confidence predictions (Figs. 2d,e and 3d–f).

In our analysis, FUGAsseM substantially extended functional annotations within the human microbiome, highlighting key ecological processes such as iron and ammonium ion homeostasis and toxin metabolism (Fig. 5b). Our predictions reveal insights into the ecological dynamics of iron within the gut, identifying specific protein families involved in its acquisition and regulation. This is particularly important given that iron, albeit essential for numerous enzymatic processes in microbial physiology, is a limiting nutrient in the gut, leading to competitive interactions both among microbial species and between microorganisms and the host^69,70^. The transportation of iron is a crucial bioprocess in the gut given that iron availability is dependent on barrier integrity and inflammation; in turn, this influences pathogen colonization and the expression of virulence factors, as well as the composition and resilience of the microbial community. Bacteria possess complex mechanisms to sequester iron from their environment, which can affect host iron homeostasis and overall health, leading to a tightly regulated balance between iron acquisition and host defense systems^111,112^.

**Extended Data Fig. 10:**
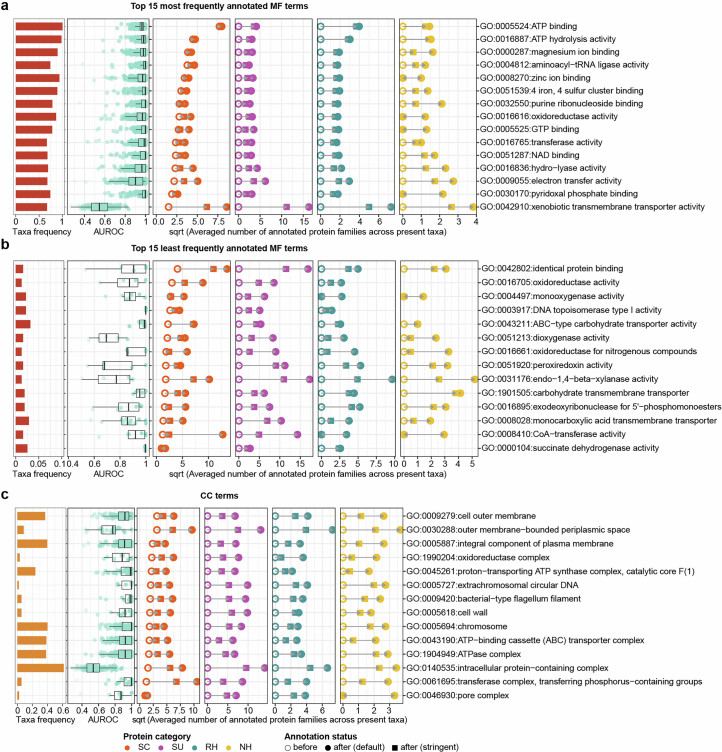
Uncharacterized proteins are predicted to be broadly distributed and species-specific MF and CC terms with high confidence in the human microbiome. (**a**) Enumeration of the fraction of annotated taxa (first column), AUROC values per taxon (middle column; *n* = 3,849 total term-species pairs for prediction), and numbers of annotations (third column) for species-shared BP terms predicted by FUGAsseM in the HMP2. The top 15 terms with the largest number of species with at least one assignment are listed in decreasing order of average preserved annotations across species before running FUGAsseM (full results in Supplementary Table 15,16). ‘Before’ represents the number of annotations before running FUGAsseM. ‘After (default)’ represents the number after running FUGAsseM with the ‘default’ threshold, and ‘after (stringent)’ reflects values based on the ‘stringent’ threshold. Box plots display the median (line at the 50th percentile), interquartile range (box spanning the 25th to 75th percentiles), and whiskers (extending to 1.5× IQR). (**b**) Fraction of annotated taxa (first column), AUROC values per taxon (middle column; *n* = 89 total term-species pairs for prediction), and numbers of annotations (third column) for species-specific MF terms predicted by FUGAsseM from the HMP2 cohort. The 15 least frequently predicted terms are listed in decreasing order of mean number of preserved annotations as in **a** (full results in Supplementary Table 17,18). (**c**) Fraction of annotated taxa (first column), AUROC values per taxon (middle column; *n* = 926 total term-species pairs for prediction), and numbers of annotations (third column) for species-shared CC terms predicted by FUGAsseM in the HMP2. CC terms are listed in decreasing order of mean number of preserved annotations as in **a** (full results in Supplementary Table 15,16). Box plots as in **a**. Source data

Similarly, our method identified proteins linked to ammonium ion metabolism, shedding light on a crucial but less explored aspect of nitrogen cycling within gut communities. The ability to efficiently process ammonium ions in certain microorganisms affects the entire community by influencing nitrogen availability, which is essential for the synthesis of critical biomolecules such as amino acids and nucleotides^113^. Nitrogen cycling, particularly through ammonium ions, is pivotal for microbial growth and ecosystem function and disruptions in this cycle can lead to notable shifts in community structure and function^114,115^. These metabolic pathways also highlight the adaptability of gut microorganisms to use ammonia under low-oxygen conditions, which is often seen in densely populated regions of the gut, contributing to the efficiency of nutrient recycling and energy conservation in this ecosystem^116,117^. Our approach further predicted proteins involved in other transport-related functions, including amino acid, anion and dicarboxylic acid transmembrane transport (Fig. 5b). These processes reflect a range of nutrient uptake and waste removal, affecting microbial viability and interaction within the gut ecosystem. We also detailed the MFs and CCs newly annotated by FUGAsseM, such as the ATP activity involved in essential microbial energy processes, species-specific functions such as endo-1,4-β-xylanase activity and various CCs (Extended Data Fig. 10). The annotation of endo-1,4-β-xylanase is a noteworthy example as it underlines the role of highly specific microorganisms in the degradation of individual plant-derived fibers within the gut, thus simultaneously influencing host and microbial nutrient processing. While our predictions suggest that certain functions are primarily associated with specific species, this may reflect either true biological exclusivity or the current limitations of available data. Future studies with expanded datasets and experimental validation will help further distinguish between species-specific and widely conserved functions.

In the process of expanding the functional landscape of the human gut microbiome, FUGAsseM’s annotations suggested several BPs that are more prevalent or critical than previously understood. For instance, the process of cell shape regulation, which influences microbial adaptability, colonization and interaction within the gut environment, was newly predicted across a diverse array of species, including both gram-positive and gram-negative bacteria. Similarly, the annotation of flagellum components in various species from different phyla highlights an unexpected prevalence of motility functions (Supplementary Table 15). These findings highlight the diversity of strategies used for microbial colonization, interaction and nutrient acquisition within the human gut.

Additionally, FUGAsseM’s annotation underscores the extent to which even well-studied taxa possess expansive pangenomes that remain undercharacterized in endogenous, human-associated communities. Among the microorganisms analyzed, *F.* *prausnitzii* and *Bacteroides massiliensis* were notably underannotated (fraction of BP annotations: 29.2% and 27.9%, respectively) (Fig. 4a). Specifically, *F.* *prausnitzii*, with diverged phylogeny and varying phenotypes^81,118^, showed considerable gaps in functional annotations (15,122 of the total 21,358 protein families were uncharacterized), likely because of its complex strain variability, which can impact MTX species-based normalization and dilute coexpression signals. The expression profiles of uncharacterized *B.* *massiliensis* proteins showed moderate correlations with characterized proteins (average Pearson’s correlation, *R* = 0.56) (Fig. 1c), which may be because of the limited number of previously annotated protein families (only 8% of *B.* *massiliensis* protein families have been annotated with informative BP terms). Interestingly, our predictions also highlight the extent to which even well-studied taxa, such as *E.* *coli* and *B.* *thetaiotaomicron*, exhibit large, undercharacterized pangenomes when found in natural, human-associated communities. In some instances, these taxa have seen dramatic expansions in their pangenome annotations, which is comparable to other highly prevalent taxa in the human gut that lack extensively studied isolate genomes, such as *Eubacterium rectale* and *H.* *hathewayi* (Fig. 4a). It is noteworthy that, even with these improvements, the limited knowledge of annotations and coexpression patterns still diminish the effectiveness of the guilt-by-association approach in FUGAsseM. Incorporating additional types of data in future studies, such as structural data, could potentially mitigate this limitation. These insights underscore the potential for unexplored biological functions within the gut microbiome, emphasizing the need for further research to fully elucidate these processes and their implications.

One example of note is FUGAsseM’s newly predicted phage defense and CRISPR–Cas proteins in *F.* *prausnitzii*, as this represents a pathway of general interest in an especially health-relevant taxon. Any predicted function assignment, whether in isolates or a community, remains putative until it is verified by guided experimentation. One potential approach to validate these might be deleting newly predicted CRISPR system elements to observe the impact on plasmid conjugation and phage infection^119^. However, *F.* *prausnitzii* is challenging to isolate from stool samples and requires specific culturing techniques that mimic the anaerobic conditions in the gut^120^. Additionally, *F.* *prausnitzii* has a complex and highly variable genome, making genetic manipulation difficult^120^. As an alternative, it may be possible to take advantage of the ‘natural experiment’ of variable *F.* *prausnitzii* genetics^7^. This would involve selection of a diverse group of *F.* *prausnitzii* strains that naturally span a combinatorial range of presence and absence patterns for predicted CRISPR–Cas systems from the gut microbiome, expose them to various phages (for example, Myroviridae and Siphoviridae) in stool culture and assess their susceptibilities. However, it is not clear that appropriate *F.* *prausnitzii* phages are available for such a screen without themselves requiring new, labor-intensive isolation. Despite the challenges posed by the organism’s isolation difficulties and *F.* *prausnitzii’*s genetic complexity, exploring the newly predicted phage defense and CRISPR–Cas proteins represents an important avenue of research, which could potentially benefit from leveraging other validation techniques such as heterologous expression and fluorescence-based assays.

While FUGAsseM is able to leverage a range of capabilities unique to microbial communities, such as community-wide MTX, it is also limited in some ways by microbiome assays. As introduced above, MTX is limited in sensitivity to rare microorganisms and transcripts and the technology itself can be difficult to apply because of the need to deplete diverse ribosomal RNAs without damaging mRNAs^121,122^. MTX-based coexpression might include more noise and lack sensitivity for low-abundance taxa or transcripts, as compared to highly controlled isolate-based experimental expression profiles, but is applicable to almost any taxon or pathway detected in a community (Fig. 2a,b). When focusing on MGX, genomic proximity can be informative for predicting functions encoded at the same locus (for example, operon). This relies on MGX assembly, which can easily break down especially in communities^123^. It also requires organisms to possess substantial operonic structure, which not all microorganisms do^124^. Even when relying on simple cross-organismal sequence homology, it is difficult to avoid circularity and ‘annotation rot’ by transferring annotations that themselves have been made solely by sequence-based transfer^125,126^, which is a challenge faced by the broad field for function prediction. Both our evaluation on predictions with new experimental evidence independent of training data and annotations with experimental evidence in the gold-standard set suggests that we have avoided this pitfall so far but it may also be useful to include additional orthogonal data types, such as protein structural similarity. Lastly, many assay types that are especially informative for single-organism function prediction are simply not yet available in microbial community contexts, such as protein–protein interactions or genetic epistasis.

However, despite these limitations, there is a need to address the drastic underannotation of protein function within microbial communities. It will remain extremely challenging to translate the health potential of the human microbiome without a deeper understanding of host–microorganism and microorganism–microorganism molecular interactions, let alone understand the ecologies of environmental microbiomes that are less well studied. This cannot be achieved when many millions of microbial proteins remain functionally uncharacterized. While experimental assays are the gold standard for such annotation, the scope of the problem means that it must be guided and accelerated computationally. FUGAsseM provides a first pass at annotating the most accessible portion of the ‘dark matter’ of microbial communities and the human microbiome and we hope that future applications and expansions of the method will improve our molecular understanding of the microbiome and the mechanisms that microorganisms use to interact with each other and their environments.

## Methods

### Overview

FUGAsseM provides a system for predicting new functional assignments for uncharacterized proteins found in microbial communities through the integration of various community-wide data types. Using a guilt-by-association approach, FUGAsseM first preprocesses each data type to generate a corresponding network representation (that is, with proteins as nodes and edges representing protein–protein functional associations suggested by that data type). Then, for each function of interest (defined as a gene set, such as a GO term), FUGAsseM trains a two-layer machine learning classifier to assign proteins to the function on the basis of their network relationships with other proteins, particularly those already assigned to the function. Here, we demonstrated FUGAsseM’s accuracy and superior coverage in the task of predicting functional assignments for proteins from the human gut microbiome in comparison to methods trained on noncommunity data, including two state-of-the-art function prediction methods. We then applied FUGAsseM to generate and analyze a catalog of new functional assignments for previously uncharacterized human gut proteins. Key aspects of the FUGAsseM methodology are summarized below, with further information on network data, machine learning approaches and evaluation procedures provided in the Supplementary Information.

### FUGAsseM methodology

FUGAsseM relies most heavily on (1) MGX to create a catalog of all potentially characterizable proteins in a set of target communities; (2) a gold-standard set with initial functional assignments (where a function is defined as a set of genes that fulfill similar roles or biochemical conversions); and (3) MTX coexpression for initial function prediction whenever possible, as it is available for many environments of interest and covers the majority of proteins when available^44,109,127^. When available, these can be integrated with other data types (homology, genomic proximity, etc.). FUGAsseM provides predicted estimates for each protein family assigned to each function of interest. It uses a two-step strategy to process network data and predict functions of microbial community protein families, respectively.

### Protein families

FUGAsseM operates on nonredundant protein sequences within stratified taxonomic bins that are compiled from target communities using metagenomic data. To generate these protein families, metagenomic shotgun sequencing data are initially processed with quality control procedures (including adaptor detection, low-quality read base trimming and potential contaminant read elimination), which can be performed using tools such as KneadData (a quality control pipeline (http://huttenhower.sph.harvard.edu/kneaddata). In this case, MGX reads are processed to yield an input gene catalog stratified by taxonomic bin, which are generated using either assembly-based approaches (assembling metagenomes, followed by nonredundant protein family construction and taxonomic assignments^7,128,129^) or reference-based approaches (aligning metagenomic reads to the pangenomes of stratified taxa, followed by a translated search performed against reference protein sequence catalogs^130,131^).

### Gold-standard set

FUGAsseM takes a gold-standard set as input to train a list of functions (that is, sets of genes that perform similar roles or chemical transformations), which is designed to be broadly applicable to any type of functions such as GO terms^51^, KEGG pathways^60^ or MetaCyc modules^61^. For a given function type, a gold-standard set is defined by a set of functional assignments, describing which protein families (as introduced above) are already known to be annotated to which function (that is, in which gene sets). Those original functional annotations can come from any source of gene sets (for example, UniProt^56^, eggNOG^132^ or KEGG^60^).

### Network data (prediction evidence)

FUGAsseM is able to incorporate any assay data of the aforementioned protein families for function prediction in microbial communities that can be modeled as a network. FUGAsseM can be trained using any collection of one or more shotgun metagenomic datasets, optionally accompanied by paired metatranscriptomes. Each type of data is typically encoded as a weighted protein–protein network, in which the semantics of each network and its weights differ by data type. This is true for coexpression (edges represent transcriptional similarity), sequence similarity (edges represent homology), genomic proximity (edges representing co-occurrence within contigs or operons) or protein interaction (edges representing interactions between proteins or protein domains). This network ensemble is fed to the machine learning module of FUGAsseM. Comprehensive details regarding the network data are available in the Supplementary Information.

### Machine learning

FUGAsseM can accept any type of network data as input and ultimately treats them uniformly in the machine learning process for function prediction. Each data type is represented as a weighted protein–protein network, where the interpretation and weighting of each network vary by data type. FUGAsseM uses a ‘layered learning’ strategy to train machine learners (Extended Data Fig. 1h). For each function in the gold standard, each network is first processed in a data-type-specific manner by the initial layer of the machine learning module, where an individual classifier is trained to learn cofunction patterns on the basis of the shared annotation of each protein pair to the function. This results in an evidence weighting behavior that learns to upweight individual data type results only when they correspond with proteins of similar function. Next, all data types are integrated by a second layer of the machine learning module that combines individual classifiers’ predictions to train an ensemble classifier for final prediction. This procedure can be carried out in two stages—training and prediction (potentially on different datasets spanning the same community types). RFs (as implemented in Python scikit-learn) are used for machine learning in FUGAsseM, a state-of-the-art method that is widely used in microbiome applications^133^, as detailed in the Supplementary Information.

### Synthetic evaluation

To assess the accuracy and reliability of FUGAsseM, we conducted two primary evaluation analyses: cross-validation and temporal hold-out validation. Using a cross-validation approach, we compared the predictions of FUGAsseM and other state-of-the-art tools against different subsets of the gold-standard set (as detailed above), which assesses the performance of these tools in annotating proteins with their prospective functions. In the temporal hold-out process, we adopted the benchmarking approach from CAFA, a community-wide effort aimed at evaluating the performance of computational methods in predicting protein function^20–22^. In the context of CAFA, the dataset is typically held out temporally, meaning that tests consist of annotations that were not available at the time when training and predictions were made (that is, proteins that have accumulated experimental validation evidence between training and evaluation). Using this approach, the temporally held-out dataset serves as an independent validation to evaluate the performance of FUGAsseM in predicting protein functions. By withholding a subset of annotations until after predictions are made, we aim to simulate real-world scenarios where new experimentally validated annotations are continuously being added over time.

### Dataset overview

We used the data from HMP2/iHMP2 (ref. ^44^) for both benchmarking and application. A gene catalog with stratified taxonomic bins was built from 1,595 available shotgun metagenomes, which comprised 1,665,223 protein families after quality control^7^. MTX reads from 800 metatranscriptomes that are paired with the MGX samples were previously quality-controlled by KneadData (version 0.7.0) with default settings (http://huttenhower.sph.harvard.edu/kneaddata)^44^, including trimming and filtering low-quality reads, removing potential human read contamination and eliminating ribosomal RNAs.

### Evaluation approaches

All methods used to evaluate the performance of FUGAsseM in this study are detailed in the Supplementary Information. These include quantitative metrics, cross-validation procedures, temporal hold-out strategies, evaluation against experimental evidence, per-data-type ablation analyses and comparisons to existing methods.

### Application to the HMP2

#### FUGAsseM analysis

##### Preparation of protein families

We applied the FUGAsseM algorithm to proteins from the human gut microbiome (as described above). To prepare protein families with stratified taxonomic bins for FUGAsseM, we assembled quality-controlled HMP2 MGX reads into metagenomic contigs using MEGAHIT (version 1.1.3)^134^. Open reading frames (ORFs) in each contig were predicted by Prodigal (version 2.6)^135^. Complete ORFs were clustered into nonredundant gene catalogs with 95% sequence identity over 90% alignment coverage using CD-HIT (version 4.7)^136^. The resulting representative amino acid translations of the nonredundant gene catalogs were further clustered into protein families requiring 90% amino acid sequence identity and 80% coverage with CD-HIT (following the same criteria used in UniRef90)^58^. Lastly, taxonomic assignment for each protein family was performed using a utility of MetaWIBELE^7^, which searches proteins against the UniRef90 database with DIAMOND^137^ and uses a guilt-by-association approach to propagate consistent taxonomic assignments of classified members to unclassified members within the same metagenomic species pangenomes^7^.

Alternatively, the protein families stratified into taxonomic bins can also be generated using reference-based approaches. For example, HUMAnN^131^, a tool to profile functions from microbial communities, identifies nonredundant protein families at species level from metagenomes. Briefly, quality-controlled MGX reads are initially screened using MetaPhlAn^131^ to detect the known species present in each sample. Then, all sample reads are aligned against the combined pangenomes of species identified in the prescreen using Bowtie2 (ref. ^138^). After the nucleotide-level search, a translated search is conducted against a protein sequence catalog based on UniRef^58^ using DIAMOND^137^ for reads that do not initially map. This yields abundance profiles of protein families (UniRef90s) organized by the species responsible for those genes.

##### Preparation of gold-standard set

We prepared a gold standard for training from GO annotations of all available HMP2 proteins in UniProt^56^ (release 2019_01). Because of its hierarchical structure, GO terms are divided into different sizes representing how many proteins are assigned to a given term within a taxon. Large terms may represent general functions of most proteins and predicting these functions would be not enough to elucidate specific functions of unknown proteins. Small terms typically represent specialized functions but too few proteins annotated with these terms would limit the prediction efficiency because of lack of training information. Thus, we constructed an informative GO set that defines a set of terms with an adequate number of annotated proteins based on the GO database (releases 2021-07-02) with FUGAsseM’s utility. Adopting the definition from previous studies^53,54^, we propagated the annotations from child terms to all their ancestor terms within the GO DAG and defined a GO term as informative term if it contained more than certain number *k* of annotated proteins (*k* = 20 in this work, although the concept of informative GO terms does not prescribe a specific *k* value) and all of its child terms contain fewer than *k* proteins. This utility selects informative terms within taxon and then combines them through union to produce a combined, nonredundant set across taxa, ensuring hierarchical independence by excluding any terms that were parent terms of others within the set. This results in a uniform set of informative terms that can be applied to all taxa while keeping taxon-specific informative terms.

##### Preparation of network input data

To prepare MTX network input data for FUGAsseM, we built a normalized MTX abundance of protein families table with taxonomic stratification using FUGAsseM’s utility. First, quality-controlled HMP2 MTX shotgun sequencing reads per sample were aligned to the MGX-based nonredundant gene catalogs (as introduced above) using Bowtie2 with default parameter settings^138^. Second, MTX count data were calculated by featureCounts^139^ with default settings, followed by summing up the number of reads mapped to all gene sequences from the same protein family into counts of the protein family itself. Third, the resulting MTX counts were first divided by the length of the representative in each protein family in kilobases, generating abundances in reads per kilobase (RPK) units. Because MTX abundance is strongly correlated with DNA abundance estimates in communities^110,140^, we used an MTX-specific procedure to normalize MTX abundance within taxon (termed as ‘taxon-specific’ scaling^141^). It normalized the RPK units of each protein family into within-taxon total-sum scaling, where per-sample MTX abundance of a protein family is divided by the total MTX abundance contributed by its source taxon. This resulted in a normalized MTX abundance table that estimates the ‘relative expression’ of protein families within taxon, compensating for variation in RNA abundance driven by underlying changes in taxonomic abundance.

Another option to prepare MTX abundance input relies on MGX-based protein families profiled by HUMAnN^131^. In this approach, HUMAnN first aligns quality-controlled MTX reads using Bowtie2 (ref. ^138^) to the sample-specific pangenomes prescreened by the paired MGX (as introduced above). Then, HUMAnN’s translated search aligns unmapped reads against UniRef90 (ref. ^58^) using DIAMOND^137^. The resulting MTX abundance of the UniRef90 families stratified by contributed species are further normalized within species using HUMAnN’s utility ‘humann_renorm_table’.

To prepare other types of network input data, we constructed UniRef50-like protein families by clustering the representatives of the MGX-based protein families using CD-HIT (version 4.7) with 50% amino acid sequence identity and 80% coverage, which were formatted as vector-based evidence encoding sequence similarity information. We obtained genomic context information for the operated protein families by extracting cocontig patterns from the previously generated metagenomic assemblies in HMP2 (ref. ^7^). Additionally, domain–domain interactions were obtained from previous annotations for the HMP2 protein families^7^, which were assigned to the corresponding families by searching protein domains against the DOMINE database^59^ (version 2.0; a database that includes both known and predicted protein domain–domain interactions).

##### Running FUGAsseM on the HMP2 data

When applying FUGAsseM to the HMP2, we excluded extremely rare taxa for analysis that contained fewer than 500 protein families detected by MTX. Given these MTX abundance profiles, MTX-based coexpression networks were predicted by calculating Pearson correlations among the protein families. We used FUGAsseM to predict GO annotations of the HMP2 protein families with parameter settings ‘--minimum-coverage 0.05 --go-mode union --go-level 20’. For a given term, its assignment to a protein family with prediction probability greater than 0.75 was delineated as a high-confidence prediction (referred to as the default threshold), which ensures retention of true positives with high confidence while incorporating new predictions. A threshold of 0.85 was also introduced as a stringent threshold to enhance precision, sustaining an adequate true positive rate but at an especially low false positive rate. This resulted in 443,549 and 292,036 protein families being labeled to 295 informative GO terms at the default and stringent thresholds, respectively.

#### eggNOG-mapper analysis

To enable a fair comparison between FUGAsseM and eggNOG-mapper, we uniformly defined the characterization levels and consistently applied them across the same set of protein families. Specifically, eggNOG-mapper was run using default parameters (*E* value < 0.001) on protein families previously processed by FUGAsseM. Results were then filtered to retain only the informative GO terms (as described above) defined consistently between methods, ensuring a rigorous comparative assessment. Then, we compared the numbers of high-confidence predictions (prediction probability ≥ 0.75 for FUGAsseM, *E* value < 0.001 for eggNOG-mapper)) for informative GO terms produced by these two methods.

#### Protein categories

We used the homology and informative BP annotations to categorize protein families. This distinction is interpreted as classification of sequence novelty and functional characterization level. Following our previous study^7^, we defined a protein family as having ‘strong homology’ if it shared the same nonredundant CD-HIT cluster as a representative protein exhibiting ≥90% amino acid sequence identity and ≥80% coverage of the longest sequence compared to known proteins in the UniRef90 (release 2019_01)^58^. We defined strong homologs to known UniRef90 proteins as SC (strong homology, characterized) if they were assigned at least one informative GO BP term^51^ by UniProtKB^56^. SNI indicates strongly homologous proteins assigned only to higher-level noninformative BP terms and SU indicates no BP annotations. UPI indicates that the homologous UniRef90 cluster was built by UniParc proteins (which lack GO annotations). Nonhomologous proteins with <25% identity or <25% coverage or no hit at all were termed as NH, whereas RH entailed modest similarity (25% ≤ identity < 90% and 25% ≤ coverage < 80%).

#### Functional enrichment

We conducted GSEA on the HMP2 protein families using the GSEA function in the clusterProfiler (version 3.10.1)^142^ R package. Two sources of GO annotations for the protein families were used for comparison: prior annotations from UniProt^56^ and predicted annotations by FUGAsseM. For each BP term from each annotation source, we ranked the protein families by their priority scores of MetaWIBELE^7^ (measuring the bioactive potential of protein families in a specific environmental or phenotypic setting of interest) in the entire gene list and calculated a GSEA enrichment score that captured the degree to which a gene set was overrepresented at the extremes (top or bottom) of the entire ranked list of genes. To assess significance, we performed 1,000 permutations for each gene set and multiple-hypothesis testing was corrected for using a Benjamini–Hochberg false discovery rate (FDR) approach with a significance threshold FDR-adjusted *P* value < 0.25.

#### Separate analysis for MTX-based coexpression

##### MTX-based coexpression patterns

To differentiate cases of a genuine lack of coexpression from instances where proteins were inadequately expressed (for example, low prevalence), we collected proteins that were detected by MTX in more than 10% of total samples, which was termed as a well-expressed set. Using these expected well-expressed proteins, we calculated their Pearson’s correlation to distinguish legitimate lack of high coexpression cases from low-expressed cases between prior characterized and uncharacterized protein families.

##### Comparison to STRING-based coexpression

We compared MTX-based coexpression profiles by themselves to the corresponding isolates’ coexpression from STRING^57^. We matched proteins between MTX-based profiles and STRING-based profiles based on common UniProt IDs and then extracted their coexpression patterns. A pair of proteins was defined as linked if their linkage score (measuring their association degree) was more than the first quartile of the maximum score in STRING. A Mann–Whitney test was used to assess the difference of MTX-based coexpression distributions between linked and unlinked proteins in isolates. To examine the relationship between species characterization or representation and similarity of MTX-based versus STRING-based coexpression networks, we defined a metric (referred to as ‘reference representation’) calculated as the percentage of proteins from a species in the HMP2 dataset that had strong homologs in UniRef90. Then, we compared the reference representation of a species to the quantitative similarity between MTX-based and STRING-based coexpression calculated by Pearson’s correlation.

##### Coexpression network

To visualize coexpression networks of interest, we selected protein families with high-confidence predictions based on the stringent threshold (that is, prediction probability ≥ 0.85) and connected families if their correlation coefficient were more than the 90th percentile value of the whole pairwise correlations of the corresponding nodes per species per term.

### Abbreviation

All abbreviations used in this study are organized in Supplementary Table 25.

### Reporting summary

Further information on research design is available in the Nature Portfolio Reporting Summary linked to this article.

## Online content

Any methods, additional references, Nature Portfolio reporting summaries, source data, extended data, supplementary information, acknowledgements, peer review information; details of author contributions and competing interests; and statements of data and code availability are available at 10.1038/s41587-025-02813-7.

## Supplementary information

Supplementary Information Supplementary Notes for results interpretation and Methods.

Reporting Summary

Supplementary Tables 1–25 Supplementary Table 1: Proportions of protein families detected by HMP2 MTX with different characterization levels. Supplementary Table 2: A list of informative GO terms defined in this study. Supplementary Table 3: Maximum MTX-based correlation coefficients between well-expressed characterized and uncharacterized proteins from the HMP2 species. Supplementary Table 4: Coexpression scores for the matched proteins detected by MTX and STRING. Supplementary Table 5: Mean AUROC across species per term of FUGAsseM based on MTX-based and STRING-based coexpression. Supplementary Table 6: Mean AUROC across species per term of FUGAsseM based on community-integrated data and STRING-integrated networks. Supplementary Table 7: Mean AUROC across terms per species of FUGAsseM based on MTX-based and STRING-based coexpression. Supplementary Table 8: Mean AUROC across terms per species of FUGAsseM based on community-integrated data and STRING-integrated networks. Supplementary Table 9: AUROC results of each method for each term within each species based on cross-validation evaluation. Supplementary Table 10: AUROC results of each method for each term within each species based on temporal hold-out evaluation. Supplementary Table 11: Prediction contribution of each data type for overall predictions. Supplementary Table 12: Prediction contribution of each data type for predictions with experimental evidence. Supplementary Table 13: Number of protein families with high-confidence predictions from the top 25 most uncharacterized species in the HMP2. Supplementary Table 14: Per-species fraction of protein families with functional annotations before and after processed by FUGAsseM-full. Supplementary Table 15: Per-species AUROC results of the top 15 most frequently predicted terms. Supplementary Table 16: Averaged fraction of protein families with the top 15 most frequently predicted terms before and after being processed by FUGAsseM-full. Supplementary Table 17: Per-species AUROC results of the top 15 least frequently predicted terms. Supplementary Table 18: Averaged fraction of protein families with the top 15 least frequently predicted terms before and after processed by FUGAsseM-full. Supplementary Table 19: Proteins predicted to GO:0071555. Supplementary Table 20: Proteins predicted to GO:0005984. Supplementary Table 21: Proteins predicted to GO:0019058 and GO:0051607 in *F.* *prausnitzii*. Supplementary Table 22: Proteins predicted to other functions in other taxa. Supplementary Table 23: Proteins predicted to GO:0044275 in *B.* *thetaiotaomicron*. Supplementary Table 24: Protein annotations of informative GO terms assigned by eggNOG-mapper. Supplementary Table 25: Abbreviations used in this study.

## Source data

Source Data Fig. 1 Values of bar plots and box plots and statistical source data.

Source Data Fig. 2 Values of box plots, violin plots, point plots, scatter plots, heatmaps and density plots and statistical source data.

Source Data Fig. 3 Values of box plots and violin plots.

Source Data Fig. 4 Values of bar plots and point plots.

Source Data Fig. 5 Values of bar plots, box plots and point plots.

Source Data Fig. 6 Values of network plots.

Source Data Extended Data Fig. 1 Values of bar plots, box plots, point plots and scatter plots and statistical source data.

Source Data Extended Data Fig. 2 Values of box plots, violin plots, point plots, scatter plots, heatmaps and density plots and statistical source data.

Source Data Extended Data Fig. 3 Values of box plots, violin plots, point plots and density plots and statistical source data.

Source Data Extended Data Fig. 4 Values of box plots, violin plots and point plots.

Source Data Extended Data Fig. 5 Values of point plots.

Source Data Extended Data Fig. 6 Values of box plots, violin plots, point plots and line plots.

Source Data Extended Data Fig. 7 Values of bar plots and point plots.

Source Data Extended Data Fig. 8 Domain annotations of each protein.

Source Data Extended Data Fig. 9 Values of bar plots and box plots and statistical source data.

Source Data Extended Data Fig. 10 Values of bar plots, box plots and point plots.

## Data Availability

Associated data generated during this study are included in the published article and its Supplementary Information. The precomputed function predictions from the previously published HMP2 data spanning 451,830 protein families (with preserved annotation and/or FUGAsseM’s predictions) and 295 target GO terms are available online (http://huttenhower.sph.harvard.edu/fugassem). All assembled metagenomic contigs, ORFs, gene families, protein families, functional profiles, taxonomic profiles and prioritized profiles of protein families related with this study are also available online (http://huttenhower.sph.harvard.edu/metawibele). Raw data of HMP2 metagenomes and metatranscriptomes were obtained from the IBDMDB website (https://ibdmdb.org). The following public databases were used: UniProt (https://www.uniprot.org/), UniRef90 (https://www.uniprot.org/uniref/), Pfam (https://pfam.xfam.org/) and DOMINE (https://manticore.niehs.nih.gov/cgi-bin/Domine). Source data are provided with this paper.

## Code availability

The open-source FUGAsseM software is available online (http://huttenhower.sph.harvard.edu/fugassem). Manuals and online tutorials describing FUGAsseM are available on GitHub (https://github.com/biobakery/fugassem). User support is provided through the bioBakery help forum (https://forum.biobakery.org). Additional software details are provided in Methods.
