# Supplementary material for: Predicting functions of uncharacterized gene products from microbial communities
Source: Nat Biotechnol. 2025 Oct 15;44(7):1126–41. doi: 10.1038/s41587-025-02813-7 (PMC13368603; doi:10.1038/s41587-025-02813-7)
Supplement: Supplementary file 1 — Supplementary Notes for results interpretation and Methods. [file 41587_2025_2813_MOESM1_ESM.pdf]

nature biotechnology

Supplementary information https://doi.org/10.1038/s41587-025-02813-7

Predicting functions of uncharacterized gene products from microbial communities

In the format provided by the authors and unedited

Table of Contents

Supplementary Notes **1**

Supplementary Note 1: FUGAsseM predicts protein functions by levigating co-expression from metatranscriptomes 1

Supplementary Methods **3**

FUGAsseM methodology 3

Network data (prediction evidence) 3

Machine learning 4

Synthetic evaluation 5

Quantitative measurements of evaluation 5

Cross-validation 6

Temporal hold-out 6

Evaluation with experimental data 6

Per-data type ablation evaluation 6

Comparison with STRING 7

Comparison with NetGO2, DeepGOPlus and the structure-based approach 7

References **8**

Supplementary Notes

Supplementary Note 1: FUGAsseM predicts protein functions by levigating co-expression from metatranscriptomes

Housekeeping and essential functions were greatly expanded by leveraging co-expression between transcripts of uncharacterized protein families and characterized target families (**Fig. 6a**). For example, the annotations of cell wall organization (GO:0071555) were annotated to diverse species (**Fig. 5a**). The cell wall provides mechanical support to the cell, allowing it to maintain its shape and resist mechanical stresses[77, 143, 144]. Multiple enzymes are responsible for bacterial cell wall biosynthesis, such as UDP-N-acetylmuramate dehydrogenases, UDP-N-acetylmuramoyl enzymes[145]. A number of *E. coli* uncharacterized protein families with strong homology to the known were predicted to the cell wall with high confidence (satisfied the “stringent” threshold). These families were strongly correlated with the UDP-N-acetylmuramoyl enzymes at expression level (**Fig. 6a; Supplementary Table 19**), indicating underlying functional associations and FUGAsseM’s ability in characterizing *E. coli*’s big pangenomes. Moreover, FUGAsseM also dramatically contributed to functional characterization of less-studied species in communities such as *Staphylococcus*, which has been reported to colonize in the human gut next to the nasopharynx and oral cavity interacting with immune cells [146, 147]. Notably, many novel proteins from *Staphylococcus sp CAG 324* were confidently predicted to be involved in cell wall organization by FUGAsseM, where MTX-based co-expression evidence achieved the strongest contribution in the prediction model (**Fig. 6a; Supplementary Table 18**), highlighting the importance of co-expression in predicting functions of novel proteins that lack sequence similarity with known proteins.

Meanwhile, the promising contribution of co-expression was also observed for predicting species-specific functions (**Fig. 6b**). For example, the disaccharide metabolic process (GO:0005984) was more uniquely predicted to a few HMP2 species (**Fig. 5b**). Disaccharides are an important source of energy for bacteria, particularly those that inhabit the human gut[78]. The bacteria’s ability to utilize disaccharides plays a significant role in their colonization and survival within the gut environment. These bacteria can hydrolyze disaccharides by a variety of disaccharidases to utilize a wide range of disaccharides present in the diet, such as tagatose and sucrose, and use the resulting monosaccharides as an energy source. Thus, the ability of utilizing disaccharides can be an important factor in bacteria colonization and survival in the gut. Uncharacterized proteins from the common gut bacteria such as *Faecalibacterium prausnitzii* and *Hungatella hathewayi* were highly predicted with the function of disaccharide metabolic process. At sequence level, these uncharacterized proteins encoded similar classes of protein domains with the characterized disaccharidases-related enzymes, while lacking notable global sequence similarity (**Supplementary Table 20**). Intriguingly, the confidently predicted new members were strongly co-expressed with the annotated disaccharidases enzymes (**Fig. 6b**), supporting the functional potential of disaccharide metabolism for the uncharacterized proteins.

The functional characterization of *Hungatella hathewayi*, another common gut taxa containing relatively small fraction of novel proteins but large fraction of known proteins without functional annotations (**Fig. 1b**), was remarkably improved by taking advantage of MTX-based co-

expression (**Fig. 6d**). *H. hathewayi* has been reported with critical associations with the host health, such as catabolizing various glycosaminoglycans contributed to the colonization in the human gut[79]. FUGAsseM assigned high-confidence functional annotations to lots of uncharacterized proteins, resulting in a better functional resolution of *H. hathewayi*. For example, chemotaxis (GO:0006935) was better annotated in *H. hathewayi*. Cell movement directed by chemotaxis and achieved by motility assists bacteria in nutrient foraging and should therefore be a fitness attribute[80]. Flagellin proteins from *H. hathewayi* might contribute to its susceptibility to penicillin, ampicillin and ceftizoxime, etc.[148]. Consistent with the characterized proteins, newly predicted chemotaxis-related proteins encoded CheW−like/flagellar motor domains (**Supplementary Table 22**), indicating functional relatedness. Their co-expression patterns further confirmed the predictions of chemotaxis to uncharacterized members (**Fig. 6d**). Likewise, some uncharacterized proteins were predicted with the cobalamin biosynthesis (GO:0009236) and strongly co-expressed with the known members (**Fig. 6d**). *H. hathewayi* encoding proteins such as cobyric acid synthase involved in cobalamin biosynthesis pathway is an efficient glycosaminoglycan-degrading species and contributes to cobalamin utility in human gut[79, 149].

Like *Bacteroides thetaiotaomicron*, the massive uncharacterized proteins of *Bacteroides fragilis* commonly found in the lower gastrointestinal tract were functionally annotated by leveraging MTX-based co-expression (**Fig. 6f**). *B. fragilis* can produce a variety of glycoside hydrolases[150], including pectinases that break down pectin, a type of polysaccharide found in plant cell walls. Another glycoside hydrolase xylanase family protein was co-expressed with another known protein Alpha−xylosidase, which is another glycoside hydrolase in family 31[89]. Like with the newly predicted glycoside hydrolase family 97 protein, which encodes Pfam domain that matches the prediction including a retaining and inverting mechanism carbohydrate active enzymes (CAZymes), was co-expressed with alpha-xylosidase. Biologically, this demonstrates that glycoside hydrolases were expressed as a set of proteins. Endo-1,4 beta-xylanase increases the degradation efficiency of forms of xylan via the creation of new xylanase binding sites[88] and was co-expressed with a newly predicted acetylxylan esterase (GO:0046555) that is supported by its UniProt annotations (**Supplementary Table 22**). Many *B. fragilis* uncharacterized proteins including novel proteins were predicted to the process of polysaccharide catabolization, where MTX-based co-expression worked as the strongest contributor in the prediction models by FUGAsseM (**Fig. 6f; Supplementary Table 22**). These extended annotations will help us better understand the key role of *B. fragilis* in breakdown of sugar for recycling energy sources for the bacterial community[151] and the enzyme activity in the colonization of *B. fragilis* in the gut microbiome.

Supplementary Methods

FUGAsseM methodology

Network data (prediction evidence)

MTX-based co-expression network

MTX network input. One MTX-based abundance table of the aforementioned protein families with stratified taxonomic bins is used as input for co-expression network construction. Metatranscriptomes are typically paired with corresponding metagenomes that provide the underlying protein family catalog for the same target communities. To get this MTX-based abundance input, users have the option to prepare it either through their own analysis or using the utility provided in the FUGAsseM package. In brief, QC'ed MTX reads (processed by eliminating adapters, trimming low-quality read bases and removing potential contaminant reads and ribosomal RNAs) are typically aligned to the MGX-based protein families with stratified taxonomy. Then, the "relative expression" of protein families within each taxon is estimated by normalizing the preliminary MTX abundances with those from its source taxon per sample. This normalization utilizes a within-taxon total-sum-scaling method, adjusting the changes of gene copy number in communities[141].

MTX network prediction. Based on the normalized MTX abundance table, taxon-specific MTX abundance data are generated using the input stratification of protein families to individual taxa. These taxon-specific expression data mimic the RNA-seq data of a single organism and thus enable us to construct co-expression networks (in which edges represent similarities in transcription) within each taxon. Several tuning parameters are considered to preprocess taxon-specific expression data. First, when appropriate, different procedures for prefiltering zeros encode different assumptions regarding biological versus technical zeros in MTX. As introduced in our previous study[110], three filtering approaches are possible, including "lenient" filtering (i.e., excluding proteins that are always zero-valued in MTX or gene-copy estimates by MGX), "semi-strict" filtering (i.e., filtering samples for a given protein if both their MTX and gene-copy estimates are zero), and "strict" filtering (i.e., filtering samples per-protein if either their MTX or gene-copy estimate are zero-valued). Second, variance-stabilizing transformations are recommended for correlation estimation based on MTX data. Log transformation is used as default after prefiltering zeros ("lenient" filtering as default), where zeros are additively smoothed by half of the smallest non-zero measurement on a per-protein basis. Finally, additional filtering procedures are included, such as excluding taxa that have very few annotated proteins (<10% by default) and pruning proteins absent in most samples (>90% by default). Based on the tuned taxon-specific normalized MTX abundance data, MTX-based networks are constructed by calculating co-expression similarity among proteins within taxon using Pearson correlations as default (other types of correlations are available). Coefficients from the correlations are formatted as features used for FUGAsseM's ML module.

Sequence homology network

Homology network input. Sequence similarity data of the aforementioned MGX-based protein families can be used as homology network input data. To get access to sequence similarity, one approach is clustering the representative sequences of the target protein families (i.e., a clustering of all non-redundant protein sequences), where the representatives could be either the longest sequence in the cluster or best-annotated member of the cluster (UniRef standard). For example, UniRef50-like protein families can be constructed by clustering the target protein families with 50% amino acid sequence identity and 80% coverage of the longest sequence in a cluster. The resulting clustering information serves as sequence similarity data for the MGX-based protein families.

Homology network prediction. Sequence similarity data can be formatted into a single-evidence vector, representing homology-based networks across taxa (where edges indicate homology). This type of network is encoded by co-clustering relationships for the functions of interest. That is, for a given cluster, if at least one protein family in the cluster is annotated to a given function from the gold standard set introduced above, the other members will be connected to this known protein family for this function. In this manner, a vector is generated for each function of interest, representing protein-over-function relationships, which indicates whether a protein family connects with the known protein families (i.e., originally assigned to this function). This vector-based network is formatted as a single feature feeding to FUGAsseM's ML module.

Genomic proximity network

Genomic co-occurrence data for the MGX-derived protein families can serve as input for the genomic proximity network (where edges indicate co-occurrence within contigs or operons). Typically, this type of evidence is encoded in the metagenomic assemblies, which provide the physical closeness or adjacency of genomic elements (e.g., genes, genomic regions) within assembled contigs or scaffolds. Genomic-proximity networks within each taxon are inferred from the relative positions of genes within contigs or scaffolds, operon structures, gene clusters, or other patterns of genomic organization[27, 28]. This genomic-proximity network is formatted as a feature matrix used for the ML process of FUGAsseM.

Physical interaction network

FUGAsseM can also use interaction network data of the aforementioned MGX-based protein families for function prediction. When interaction annotations (e.g., protein-protein interactions[34] or protein domain-domain interactions[59]) of the protein families are available, an interaction network can be constructed based on the co-annotation patterns among protein families within taxon. In these networks, two protein families are linked if they participate in the same interaction. This type of network data is organized into a feature matrix feeding to FUGAsseM's ML module.

<u>Machine learning</u>

Training an individual classifier for each network input

Our application here is straightforward: an ensemble binary classifier is used for providing estimates of class probabilities of function assignment. In the first layer of the ML process, individual RF classifiers are trained using each type of network data for each function from the

gold standard. In this procedure, we employ stratified cross-validation to train classifiers, ensuring that folds maintain the proportion of proteins for each function class. In each classifier, proteins are initially divided randomly into *k* subsets (folds, five as default) of equal size. A distinct subset is designated for testing the model, while the remaining k−1 subsets are utilized for training. Protein labels (based on the gold standard) are randomly balanced before training, which ensures each fold contains a balanced proportion of positive and negative cases. For each function (gene set) of interest, a RF classifier utilizes the training data to learn the relationship between the features and the target classes. This process is iterated k times until each of the k subsets used once as the testing set.

Training an ensemble classifier for network integration

For a given function, the resulting predicted relationships from individual RF classifiers in the first layer are fed to an additional classifier in the second layer of ML. During this process, the predictions of data-type-specific classifiers are treated as features for an ensemble RF classifier. This classifier aggregates the predicted relationships from each network with assigned weights, which robustly integrates all types of network data and captures the heterogeneity across data while maximizing classification accuracy. The same cross-validation strategy used in the first layer is employed to train ensemble RF classifiers based on the gold standard.

Making predictions after training

The resulting RF classifiers from the training procedure are used to predict probability estimates for assigning functions to all proteins of interest. For within-dataset prediction (i.e., training and predicting within the dataset), the dataset is randomly split into *k* groups. One group serves as the test set while the remaining groups form the training set, which is repeated *k* times. For each function of interest, RF classifiers trained with the training sets and gold standard are employed to predict new proteins coming from the test sets. For cross-dataset prediction (different datasets spanning the same community types), one dataset is utilized for training RF models while the other is used for prediction. Ultimately, the predicted assignment probability of each protein family to each function is reported as FUGAsseM's output.

Synthetic evaluation

<u>Quantitative measurements of evaluation</u>

We evaluated tools' performance for function prediction in this study by quantifying statistical power and prediction accuracy. Following the lead of the CAFA experiments[20-22], we calculated the quantitative measurements using sliding thresholds of prediction probability. For a given functional term *f* and decision threshold *t*, (i) when a protein family *x* has a prediction probability greater than or equal to threshold *t,* it is considered as predicted positive. In this case, if protein family *x* is also initially annotated with function *f* in the gold standard, it is treated as a true positive (TP), and otherwise protein family *x* is a false positive (FP); (ii) when the prediction probability of protein family *x* is less than threshold *t*, *x* is considered as predicted negative, where *x* would be false negative (FN) if it is in the set of proteins originally annotated with function *f* and otherwise *x* is true negative (TN). Based on these definitions, the sensitivity (also called recall), specificity, and precision for a given function *f* under threshold *t* are calculated using standard definitions.

The area under the ROC curve (AUROC) of function *f* was calculated over all protein families for the varying prediction threshold. Standard False Discovery Rate (FDR) and False Positive Rate (FPR) were also used to evaluate prediction accuracy.

Cross-validation

Unless specified otherwise, all classifiers were evaluated by a cross-validation approach based on the gold standard set that were used for training models, as introduced above. In our analysis, the within-dataset prediction capability was evaluated through five-fold cross-validation. The dataset was randomly divided into five groups with equal size. One of the groups was designated as the test set, while the remaining groups served as the training set. The model underwent training using the training set and evaluation on the test set five times independently, ensuring that each group served as the test set once.

Temporal hold-out

Similar to the temporal hold-out approach in CAFA, we built a ground-truth set to evaluate FUGAsseM's new annotations that have not been seen at the time of training. First, we chose an earlier release version of UniProt[56] (i.e., T_0: release 2019_01) as the first time point, GO annotations from which were used for training by FUGAsseM. Then, we chose a newer version of UniProt released in January 2022 (i.e., T_1: release 2022_01) as the second time point. We collected new annotations that have accumulated experimental evidence during the period from T_0 to T_1 as a ground-truth dataset. GO annotations with evidence codes EXP, IDA, IPI, IMP, IGI, IEP, TAS, and IC in UniProt were considered as verified experimental validation. For each GO term in the ground-truth set, if a protein family with high-confidence prediction was newly annotated to this term or its descendent terms, this protein family was treated as a positive example; if this protein family was not validated in the ground-truth set, it was treated as a negative example.

Evaluation with experimental data

To assess the robustness of FUGAsseM and evaluate the potential impact of homology-based circularity, we conducted an additional validation using only experimentally confirmed annotations from our gold standard dataset. Using cross-validation, we re-assessed FUGAsseM by restricting the evaluation to annotations with prior experimental validation. Performance metrics were recalculated separately for protein families with and without experimental evidence to quantify any systematic differences. Additionally, we examined the contribution of MTX-derived co-expression features to functional predictions by comparing their relative importance in models trained on experimentally validated annotations versus those trained on non-validated annotations.

Per-data type ablation evaluation

To assess the contribution of individual evidence types to function prediction, we conducted an ablation study by systematically training FUGAsseM models while removing one feature type at a time. For robust evaluation and to minimize potential circularity from homology-based annotations, we employed a temporal hold-out validation approach, ensuring that predictions

were made independently of prior database annotations. Each model variant was trained using the remaining evidence types and evaluated against the ground-truth set. The impact of each data type was quantified by comparing model performance across GO aspects.

Comparison with STRING

We assessed FUGAsseM's performance on community-based data by comparing it with existing isolate-based data. To this end, we downloaded isolate-based network data from STRING[57] ("Search Tool for the Retrieval of Interacting Genes/Proteins", a biological database of functional protein association networks) version 11.5 on January 01, 2022. For species detected in the human gut microbiome (see "Application to the HMP2" section below), we applied FUGAsseM in the default mode using community-based data and isolate-based data found in STRING, respectively. Community-based data were derived from the human gut microbiome, including co-expression from MTX, genomic proximity in metagenomic assemblies, protein sequence similarity from UniRef50-like groups, and predicted domain-domain interactions (see "Application to the HMP2" section below). Averaged AUROC values for each GO term across species (or each species across GO terms) were calculated based on the prediction generated by community-based data and isolate-based data respectively, where the input GO annotations from the gold standard were used as true labels for AUROC calculation. That means, if a protein family with high-confidence prediction was originally annotated, this protein family was treated as a positive example; otherwise, it was treated as a negative example. Pearson's correlation was used to compare matched terms or species between community-based predictions and isolate-based predictions.

Comparison with NetGO2, DeepGOPlus and the structure-based approach

The performance of FUGAsseM was also evaluated by comparing with other single-organism methods. We benchmarked two state-of-the-art tools NetGO2.0[62] (released on September 20, 2020) and DeepGOPlus[63] (version 1.0.1), both of which were reported as top-performing tools in CAFA[21, 22]. Limited by the implementation of these tools, we performed the top ten most abundant species in the human gut microbiome[7] using DeepGOPlus and top five species using NetGO2.0 (due to the limited throughput of its web-server based implementation) with default settings. To ensure a rigorous and standardized evaluation, predicted GO terms were compared to the gold standard set by mapping predictions to the same level of annotation granularity. Specifically, we propagated annotations using the GO hierarchy, ensuring that parent-child relationships were consistently accounted for in both the predicted and gold standard annotations. This step minimized discrepancies arising from different levels of annotation specificity across methods.

Following the approach outlined previously[64], we implemented a structure-based approach for function prediction by systematically assessing structural similarity to proteins with known functions from the gold standard set. As structure prediction at large scale is computationally intensive, we prioritized HMP2 novel protein families (NH) presented in our previous publication[7] and utilized existing UniProtKB structures for known families. Specifically, we first predicted structures of NH families using AlphaFold2 implemented through ColabFold[65], resulting in 3,007 reliable protein structures. High-confidence structures were defined as those achieving a Predicted Local Distance Difference Test (pLDDT) score ≥70, as previously recommended in

literature[66]. Meanwhile, we retrieved all available structures for UniRef90 families with strong homology to those in this study from UniProtKB, yielding a dataset of 443,389 additional structures. Subsequently, we compared these reliable structures to known functional references by performing structural alignments using Foldseek[67] with parameter settings “-e 0.001 -s 9.5”. Functional annotations for each protein family were assigned based on structural similarity (i.e., TM-score, a metric used to assess the similarity between two protein structures) to its best-aligned annotated counterpart.

For AUROC calculation, as described in the “Quantitative measurements of evaluation” section above, we ranked predicted GO terms based on their confidence scores assigned by each method. The AUROC metric was then computed by assessing the true positive rate (sensitivity) against the false positive rate at varying classification thresholds. True positive cases were defined as GO terms correctly recovered in the gold standard set, while false positives were defined as predictions not present in the gold standard. To ensure consistency, AUROC scores were calculated independently for each GO term per species, providing an interpretable measure of model performance across different functional categories. This approach allowed for a fair and robust comparison of FUGAsseM against alternative functional annotation methods.

References

143. Huang, K.C., Mukhopadhyay, R., Wen, B., Gitai, Z. & Wingreen, N.S. Cell shape and cell-wall organization in Gram-negative bacteria. *Proc Natl Acad Sci U S A* **105**, 19282-19287 (2008).
144. Jukič, M., Rožman, K., Sova, M., Barreteau, H. & Gobec, S. Anthranilic Acid Inhibitors of Undecaprenyl Pyrophosphate Synthase (UppS), an Essential Enzyme for Bacterial Cell Wall Biosynthesis. *Front Microbiol* **9**, 3322 (2018).
145. Deva, T., Baker, E.N., Squire, C.J. & Smith, C.A. Structure of Escherichia coli UDP-N-acetylmuramoyl:L-alanine ligase (MurC). *Acta Crystallogr D Biol Crystallogr* **62**, 1466-1474 (2006).
146. Chiba, M. et al. Staphylococcus aureus in inflammatory bowel disease. *Scand J Gastroenterol* **36**, 615-620 (2001).
147. Raineri, E.J.M., Altulea, D. & van Dijl, J.M. Staphylococcal trafficking and infection-from 'nose to gut' and back. *FEMS Microbiol Rev* **46** (2022).
148. Randazzo, A., Kornreich, A. & Lissoir, B. A Clostridium hathewayi isolate in blood culture of a patient with an acute appendicitis. *Anaerobe* **35**, 44-47 (2015).
149. Centanni, M., Sims, I.M., Bell, T.J., Biswas, A. & Tannock, G.W. Sharing a β-Glucan Meal: Transcriptomic Eavesdropping on a Bacteroides ovatus-Subdoligranulum variabile-Hungatella hathewayi Consortium. *Appl Environ Microbiol* **86** (2020).
150. Berkhout, M.D., Plugge, C.M. & Belzer, C. How microbial glycosyl hydrolase activity in the gut mucosa initiates microbial cross-feeding. *Glycobiology* **32**, 182-200 (2022).
151. Moynihan, P.J. et al. The hydrolase LpqI primes mycobacterial peptidoglycan recycling. *Nat Commun* **10**, 2647 (2019).
