# Supplementary material for: Predicting functions of uncharacterized gene products from microbial communities
Source: Nat Biotechnol. 2025 Oct 15;44(7):1126–41. doi: 10.1038/s41587-025-02813-7 (PMC13368603; doi:10.1038/s41587-025-02813-7)
Supplement: Supplementary file 2 — Reporting Summary [file 41587_2025_2813_MOESM2_ESM.pdf]

nature portfolio

Corresponding author(s): Yancong Zhang, Eric A. Franzosa & Curtis Huttenhower

Last updated by author(s): Aug 1, 2025

Reporting Summary

Nature Portfolio wishes to improve the reproducibility of the work that we publish. This form provides structure for consistency and transparency in reporting. For further information on Nature Portfolio policies, see our Editorial Policies and the Editorial Policy Checklist.

Statistics

For all statistical analyses, confirm that the following items are present in the figure legend, table legend, main text, or Methods section.

n/a	Confirmed	
☐	☒	The exact sample size (*n*) for each experimental group/condition, given as a discrete number and unit of measurement
☐	☒	A statement on whether measurements were taken from distinct samples or whether the same sample was measured repeatedly
☐	☒	The statistical test(s) used AND whether they are one- or two-sided<br>*Only common tests should be described solely by name; describe more complex techniques in the Methods section.*
☐	☒	A description of all covariates tested
☐	☒	A description of any assumptions or corrections, such as tests of normality and adjustment for multiple comparisons
☐	☒	A full description of the statistical parameters including central tendency (e.g. means) or other basic estimates (e.g. regression coefficient) AND variation (e.g. standard deviation) or associated estimates of uncertainty (e.g. confidence intervals)
☐	☒	For null hypothesis testing, the test statistic (e.g. *F*, *t*, *r*) with confidence intervals, effect sizes, degrees of freedom and *P* value noted<br>*Give P values as exact values whenever suitable.*
☒	☐	For Bayesian analysis, information on the choice of priors and Markov chain Monte Carlo settings
☒	☐	For hierarchical and complex designs, identification of the appropriate level for tests and full reporting of outcomes
☐	☒	Estimates of effect sizes (e.g. Cohen's *d*, Pearson's *r*), indicating how they were calculated

Our web collection on statistics for biologists contains articles on many of the points above.

Software and code

Policy information about availability of computer code

Data collection	No software was use.
Data analysis	Predictions of micorial gene products in microbiomes: FUGAsseM v0.3.6 developed in this study (http://huttenhower.sph.harvard.edu/fugassem)<br>Metagenomic assembly and gene catalog construction: MEGAHIT v1.1.3; Prokka v1.14-dev; Prodigal v2.6; USEARCH v9.0.2132_i86linux64; Bowtie2 v2.3.2; SAMtools v1.9; featureCounts v1.6.2<br>Protein families: Diamond v0.9.24; CD-HIT v4.7; InterProScan v5.31-70; SignalP v4.1; TMHMM v2.0; Phobius v1.01; PSORTb v3.0; MSPminer v2; MaAsLin2 v0.99.12; MetaWIBELE v0.4.4; AnADAMA2 v0.7.4<br>Functional enrichment: clusterProfiler v3.10.1<br>Data analysis and visualization: R v3.6.1 (main packages: ggplot2, ggpubr, corrplot, vegan, heatmap, gggenes, ggtree)

For manuscripts utilizing custom algorithms or software that are central to the research but not yet described in published literature, software must be made available to editors and reviewers. We strongly encourage code deposition in a community repository (e.g. GitHub). See the Nature Portfolio guidelines for submitting code & software for further information.

Data

Policy information about availability of data

All manuscripts must include a data availability statement. This statement should provide the following information, where applicable:

- Accession codes, unique identifiers, or web links for publicly available datasets
- A description of any restrictions on data availability
- For clinical datasets or third party data, please ensure that the statement adheres to our policy

Associated data generated during this study are included in the published Article and its Supplementary Tables. The pre-computed function predictions from the previously-published HMP2 data spanning 451,830 protein families and 295 target GO terms are available at http://huttenhower.sph.harvard.edu/fugassem. All assembled metagenomic contigs, ORFs, gene families, protein families, functional profiles, taxonomic profiles, and prioritized profiles of protein families related with this study are available at http://huttenhower.sph.harvard.edu/metawibele. Raw data of HMP2 metagenomes, and metatranscriptomes were obtained from IBDMDB website (https://ibdmdb.org). The following public databases were used: UniProt (https://www.uniprot.org/), UniRef90 (https://www.uniprot.org/uniref/), Pfam (https://pfam.xfam.org/), and DOMINE (https://manticore.niehs.nih.gov/cgi-bin/Domine)

Research involving human participants, their data, or biological material

Policy information about studies with human participants or human data. See also policy information about sex, gender (identity/presentation), and sexual orientation and race, ethnicity and racism.

Reporting on sex and gender	The sex and gender description was presented in Lloyd-Price, et al. Nature 569.7758 (2019): 655-662.
Reporting on race, ethnicity, or other socially relevant groupings	The description of race, ethnicity, or other socially relevant groupings was presented in Lloyd-Price, et al. Nature 569.7758 (2019): 655-662.
Population characteristics	Population characteristics were presented in Extended Data Table 1 in Lloyd-Price, et al. Nature 569.7758 (2019): 655-662.
Recruitment	The recruitment description was presented in Lloyd-Price, et al. Nature 569.7758 (2019): 655-662.
Ethics oversight	According to Lloyd-Price, et al. Nature 569.7758 (2019): 655-662, the HMP2 cohort was reviewed by the Institutional Review Boards at each sampling site: overall Partners Data Coordination (IRB #2013P002215); MGH Adult cohort (IRB #2004P001067); MGH Paediatrics (IRB #2014P001115); Emory (IRB #IRB00071468); Cincinnati Children's Hospital Medical Center (2013- 7586); and Cedars-Sinai Medical Center (3358/CR00011696).

Note that full information on the approval of the study protocol must also be provided in the manuscript.

Field-specific reporting

Please select the one below that is the best fit for your research. If you are not sure, read the appropriate sections before making your selection.

☒ Life sciences ☐ Behavioural & social sciences ☐ Ecological, evolutionary & environmental sciences

For a reference copy of the document with all sections, see nature.com/documents/nr-reporting-summary-flat.pdf

Life sciences study design

All studies must disclose on these points even when the disclosure is negative.

Sample size	Metagenomics (MGX) analysis was performed on 1,595 samples from the longitudinal HMP2 cohort (Lloyd-Price et al., Nature 569.7758 (2019): 655-662), which included individuals with varying disease activity in IBD (non-IBD control, dysbiotic CD, non-dysbiotic CD, dysbiotic UC, non-dysbiotic UC) and different ages. This analysis was used to construct gene catalogs in human microbiomes and extract protein sequence information for functional inference. Additionally, paired metatranscriptomes (MTX) from 800 samples corresponding to the same HMP2 subjects provided independent evidence to predict functions. Thus, using the HMP2 cohort was sufficient to investigate the landscape of microbial gene products and identify their potential functions. No additional statistical methods were used to predetermine sample size; the entire MGX and MTX data collection from the published HMP2 study was used. Sample sizes were consistent with pre-existing HMP2 data, where they were determined based on the magnitude and consistency of measurable differences to ensure statistical power and biological significance.
Data exclusions	The analyzed datasets were reported in a previously published paper, where samples were filtered based on data type-specific quality control measures (Lloyd-Price et al., Nature 569.7758 (2019): 655-662). For HMP2 metagenomes and metatranscriptomes, samples were required to have more than 1 million reads and at least one species detected by MetaPhlAn2.
Replication	For microbial multi-omics data analysis, all data and source codes for the computational tools used are publicly available, allowing our analysis to be reproduced using our methods or re-analyzed using other methods. Whenever possible, we reference existing literature that supports our findings. As previously reported (Lloyd-Price et al., Nature 569.7758 (2019): 655-662), multiple pilot studies and technical replicates covering a subset of samples are included in the analyzed datasets. These data were successfully integrated into the analyses of this study, ensuring that data generation methods produced reproducible results.

Randomization	In the HMP2 cohort, participants were recruited into three disease groups based on availability at each recruitment site. The detailed randomization process was previously reported (Lloyd-Price et al., Nature 569.7758 (2019): 655-662): Experimental groups could not be randomized as they depended on diagnosis. Participants were assigned to these groups according to their diagnosis. Upon enrollment, an initial colonoscopy was performed to determine the study strata. Subjects not diagnosed with IBD based on endoscopic and histopathologic findings were classified as "non-IBD" controls. This group included healthy individuals presenting for routine screening and those with more benign or non-specific symptoms. Thus, the control group, while not entirely "healthy," differs from the IBD cohorts specifically by clinical IBD status.
Blinding	In HMP2 cohort, as reported previously (Lloyd-Price, et al. Nature 569.7758 (2019): 655-662), samples were collected by clinical staff who were not blinded, as they needed to examine patients to determine their experimental group allocation. However, all data were generated by investigators who were blinded to the metadata. After data generation, computational analysis was performed using all necessary clinical information to compare the groups.

Reporting for specific materials, systems and methods

We require information from authors about some types of materials, experimental systems and methods used in many studies. Here, indicate whether each material, system or method listed is relevant to your study. If you are not sure if a list item applies to your research, read the appropriate section before selecting a response.

Materials & experimental systems

n/a	Involved in the study
☒	☐ Antibodies
☒	☐ Eukaryotic cell lines
☒	☐ Palaeontology and archaeology
☒	☐ Animals and other organisms
☒	☐ Clinical data
☒	☐ Dual use research of concern
☒	☐ Plants

Methods

n/a	Involved in the study
☒	☐ ChIP-seq
☒	☐ Flow cytometry
☒	☐ MRI-based neuroimaging

Plants

Seed stocks	N/A
Novel plant genotypes	N/A
Authentication	N/A
